# Stringent Expression Control of Pathogenic R-body Production in Legume Symbiont *Azorhizobium caulinodans*

**DOI:** 10.1128/mBio.00715-17

**Published:** 2017-07-25

**Authors:** Jun-ichi Matsuoka, Fumiko Ishizuna, Keigo Kurumisawa, Kengo Morohashi, Tetsuhiro Ogawa, Makoto Hidaka, Katsuharu Saito, Tatsuhiro Ezawa, Toshihiro Aono

**Affiliations:** a Biotechnology Research Center, The University of Tokyo, Tokyo, Japan; b Graduate School of Agricultural and Life Sciences, The University of Tokyo, Tokyo, Japan; c Faculty of Science and Technology, Tokyo University of Science, Tokyo, Japan; d Faculty of Agriculture, Shinshu University, Matsumoto, Nagano, Japan; e Graduate School of Agriculture, Hokkaido University, Sapporo, Hokkaido Prefecture, Japan; University of California, Berkeley

**Keywords:** R body, legume, pathogenesis, *reb* gene, rhizobia, symbiosis

## Abstract

R bodies are insoluble large polymers consisting of small proteins encoded by *reb* genes and are coiled into cylindrical structures in bacterial cells. They were first discovered in *Caedibacter* species, which are obligate endosymbionts of paramecia. *Caedibacter* confers a killer trait on the host paramecia. R-body-producing symbionts are released from their host paramecia and kill symbiont-free paramecia after ingestion. The roles of R bodies have not been explained in bacteria other than *Caedibacter*. *Azorhizobium caulinodans* ORS571, a microsymbiont of the legume *Sesbania rostrata*, carries a *reb* operon containing four *reb* genes that are regulated by the repressor PraR. Herein, deletion of the *praR* gene resulted in R-body formation and death of host plant cells. The *rebR* gene in the *reb* operon encodes an activator. Three PraR binding sites and a RebR binding site are present in the promoter region of the *reb* operon. Expression analyses using strains with mutations within the PraR binding site and/or the RebR binding site revealed that PraR and RebR directly control the expression of the *reb* operon and that PraR dominantly represses *reb* expression. Furthermore, we found that the *reb* operon is highly expressed at low temperatures and that 2-oxoglutarate induces the expression of the *reb* operon by inhibiting PraR binding to the *reb* promoter. We conclude that R bodies are toxic not only in paramecium symbiosis but also in relationships between other bacteria and eukaryotic cells and that R-body formation is controlled by environmental factors.

## IMPORTANCE

*Caedibacter* species, which are obligate endosymbiotic bacteria of paramecia, produce R bodies, and R-body-producing endosymbionts that are released from their hosts are pathogenic to symbiont-free paramecia. Besides *Caedibacter* species, R bodies have also been observed in a few free-living bacteria, but the significance of R-body production in these bacteria is still unknown. Recent advances in genome sequencing technologies revealed that many Gram-negative bacteria possess *reb* genes encoding R-body components, and interestingly, many of them are animal and plant pathogens. *Azorhizobium caulinodans*, a microsymbiont of the tropical legume *Sesbania rostrata*, also possesses *reb* genes. In this study, we demonstrate that *A. caulinodans* has ability to kill the host plant cells by producing R bodies, suggesting that pathogenicity conferred by an R body might be universal in bacteria possessing *reb* genes. Furthermore, we provide the first insight into the molecular mechanism underlying the expression of R-body production in response to environmental factors, such as temperature and 2-oxoglutarate.

## INTRODUCTION

R bodies are bacterial inclusion bodies and are large proteinaceous ribbons that are coiled into cylindrical structures. R bodies were first observed in *Caedibacter* species, which are obligate endosymbiotic bacteria that inhabit paramecia (1). Paramecia that harbor R-body-producing *Caedibacter* cells are referred to as killer paramecia and release bacterial cells via the cytopyge. Subsequently, sensitive nonendosymbiont paramecia are killed following ingestion of the released bacteria (2), conferring the “killer trait” of paramecia (3). R bodies play a major role in this trait, because paramecia harboring mutant *Caedibacter* strains that are defective in R body production do not express the killer trait (4).

The genes involved in R-body production were originally identified in *Caedibacter taeniospiralis* (5) and include *rebA*, *rebB*, *rebC*, and *rebD* (5, 6). Moreover, genes that are homologous to *rebA*, *rebB*, and *rebD* of *C. taeniospiralis* have been found in species of the phylum *Proteobacteria* and in *Kordia algicida* OT-1 (7), which belongs to the phylum *Bacteroidetes*, whereas no *rebC*-homologous genes have been identified in bacteria other than *C. taeniospiralis* (8, 9). It is hypothesized that *reb-*homologous genes were passed on by horizontal gene transfer—i.e., by phages or plasmids (2, 8, 10, 11). Although many bacterial species that carry *reb-*homologous genes are pathogenic to plants and animals (e.g., *Xanthomonas axonopodis* pv. *citri*, *Stenotrophomonas maltophilia*, *Burkholderia pseudomallei*, and so on) (8), the rhizobium *Azorhizobium caulinodans* ORS571, a mutualistic microsymbiont of the tropical legume *Sesbania rostrata*, possesses *reb-*homologous genes (8, 12). To date, *reb*-homologous genes have not been found in rhizobia other than *A. caulinodans* ORS571.

*A. caulinodans* ORS571 fixes atmospheric nitrogen in free-living and symbiotic states (13–15) and forms nitrogen-fixing nodules at the sites of adventitious root primordia on roots and stems. Bacteria enter stems via fissures at root primordia and colonize cortical infection pockets (16). From these infection pockets, infection threads guide the bacteria toward the nodule meristematic zone, where they are released into host cells and surrounded by plant-derived peribacteroid membranes (16). Subsequently, infected host cells are filled with differentiated bacteroids and infected areas enlarge with nodule maturation (15, 16).

The *A. caulinodans* ORS571 strain has a gene cluster containing four *reb*-homologous genes (8, 12) that are strongly suppressed by PraR, which is a conserved transcription factor among *Alphaproteobacteria* (8). In a previous study, stem nodules harboring *A. caulinodans praR* mutants that expressed high levels of *reb* genes were defective in nitrogen fixation (8). Furthermore, *praR* knockout in these nodules altered the interactions between these bacteria and their host cells according to two distinctly abnormal patterns. Specifically, host cells maintained normal shapes, and the bacteria disappeared with increasing host vacuolar sizes, or bacteria predominantly occupied host cells that had shrunken gradually (8). These observations suggest that derepression of *reb* genes at least partially reverts pathogenic traits for the symbiont. The regulatory mechanism underlying the expression of *reb* genes, however, is yet to be elucidated.

In *Caedibacter* spp., *reb* genes are responsible for the production of R bodies, which likely mediate eukaryotic cell death (4, 5). Although *reb* genes have been identified in many pathogenic bacteria, pathogenic roles of R bodies have not been directly demonstrated. On the other hand, observations of disordered intracellular symbiosis in plant hosts following derepression of *reb* genes in *A. caulinodans* suggest that *A. caulinodans* kills host cells by producing R bodies. Herein, we investigated the regulatory mechanism of *reb* gene expression in *A. caulinodans* and defined the roles of R bodies in *reb*-associated pathogenic traits, with particular emphasis on transcription repressor-activator interactions.

## RESULTS

### Genetic organization of the *reb* operon.

**FIG 1:**
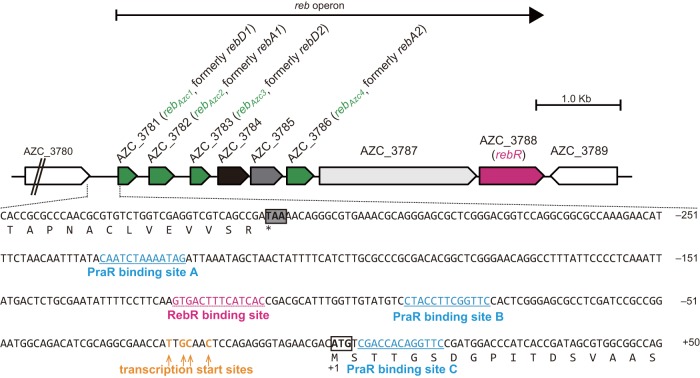
Genetic organization of the *reb* operon on the chromosome of *A. caulinodans* ORS571. The nucleotide sequence below the map shows the promoter region of the *reb* operon. Deduced amino acid sequences of the C terminus of the AZC_3780 protein and the N terminus of the Reb_AZC1_ protein are shown under their corresponding nucleotide sequences, respectively. The stop codon of AZC_3780 and the start codon of *reb*_*AZC1*_ are marked by gray and open boxes, respectively. The three PraR binding sites and the RebR binding site are underlined. Transcription start sites of the *reb* operon are indicated by arrows.

The *reb* operon includes genes from AZC_3781 to AZC_3788 on the genome of *A. caulinodans* ORS571 (Fig. 1), and transcription units and start sites were determined using reverse transcription-PCR (RT-PCR) and primer extension analyses (see Fig. S1 in the supplemental material). A comprehensive phylogenetic analysis revealed that the proteins encoded by *reb* genes in *A. caulinodans* did not belong to clusters, including RebA, RebB, and RebD of *C. taeniospiralis* (see Fig. S2 in the supplemental material). Accordingly, the *reb*-homologous genes (AZC_3781, AZC_3782, AZC_3783, and AZC_3786) *rebD1*, *rebA1*, *rebD2*, and *rebA2* (8) were renamed *reb*_*AZC1*_, *reb*_*AZC2*_, *reb*_*AZC3*_, and *reb*_*AZC4*_, respectively. An InterProScan analysis revealed that the AZC_3788 gene, which was designated *rebR*, encoded a putative transcription factor of the cyclic AMP receptor protein-fumarate and nitrate reduction regulator (Crp-Fnr) superfamily (17). The AZC_3784, AZC_3785, and AZC_3787 genes had no similarities to known *reb* genes, although BLASTp searches identified AZC_3784 homologues in *Rhizobium* sp. strain AAP43, *Oceanicaulis* sp. strain HTCC2633, and *Oceanicaulis alexandrii* DSM 11625, as well as an AZC_3787 homologue in *Inquilinus limosus*. However, no AZC_3785 homologues were found in the database.

10.1128/mBio.00715-17.1 FIG S1  Determination of transcription units and start sites of the *reb* operon in *Azorhizobium caulinodans* ORS571. (A) Map of the *reb* operon of *A. caulinodans* with target regions and primer positions for RT-PCR and primer extension analyses. (B) RT-PCR analysis to determine transcription units of the *reb* operon. cDNA was synthesized using total RNA (500 ng) from WT and Δ*praR* strains grown in BD medium at 38°C. Subsequent PCRs were performed using the 20-fold-diluted cDNAs and WT genomic DNA (1 × 10^−1^ ng µl^−1^) as the templates with the indicated primer pairs. The result showed that AZC_3781 to AZC_3788 were transcribed in a polycistronic manner, indicating that they form an operon. (C) Primer extension analysis to determine transcription start sites of the *reb* operon. Total RNA isolated from the WT and Δ*praR* strains was reverse transcribed using the SuperScript III (Invitrogen) with a fluorescein isothiocyanate (FITC)-labeled primer (P14_FITC). The sequencing reaction for ladders was performed using the Thermo Sequenase primer cycle sequencing kit (GE Healthcare) with the P14_FITC primer and the DNA fragment that was amplified by PCR from WT genomic DNA using primer pair P1-P2. Both primer extension products and sequencing reaction mixtures were electrophoresed in denaturing 6% polyacrylamide gels containing 8 M urea. Gels were scanned for FITC fluorescence using an FLA3000 system (Fujifilm). Transcription start sites are indicated by arrows in the resulting image. This result shows that the *reb* operon is transcribed from 25, 23, 22, and 19 bp upstream of the AZC_3781 start codon. Download FIG S1, PDF file, 0.2 MB. Copyright © 2017 Matsuoka et al. 2017 Matsuoka et al. This content is distributed under the terms of the Creative Commons Attribution 4.0 International license.

10.1128/mBio.00715-17.2 FIG S2  Phylogenic relationships among proteins encoded by *reb*-homologous genes. BLASTp analyses were performed using deduced amino acid sequences encoded by *rebA*, *rebB*, and *rebD* on the pKAP298 plasmid of *Caedibacter taeniospiralis* and AZC_3781, AZC_3782, AZC_3783, and AZC_3786 of *A. caulinodans* ORS571 as queries (E < 0.01) in the National Center for Biotechnology Information (NCBI) server (http://www.ncbi.nlm.nih.gov/BLAST/). The resulting sequences were combined, and phylogenic analyses were performed using MEGA5 software (K. Tamura, D. Peterson, N. Peterson, G. Stecher, M. Nei, S. Kumar, Mol Biol Evol 28:2731–2739, 2011, https://doi.org.10.1093/molbev/msr121). Download FIG S2, PDF file, 0.2 MB. Copyright © 2017 Matsuoka et al. 2017 Matsuoka et al. This content is distributed under the terms of the Creative Commons Attribution 4.0 International license.

### Contributions of the *reb* operon to R-body formation.

**FIG 2:**
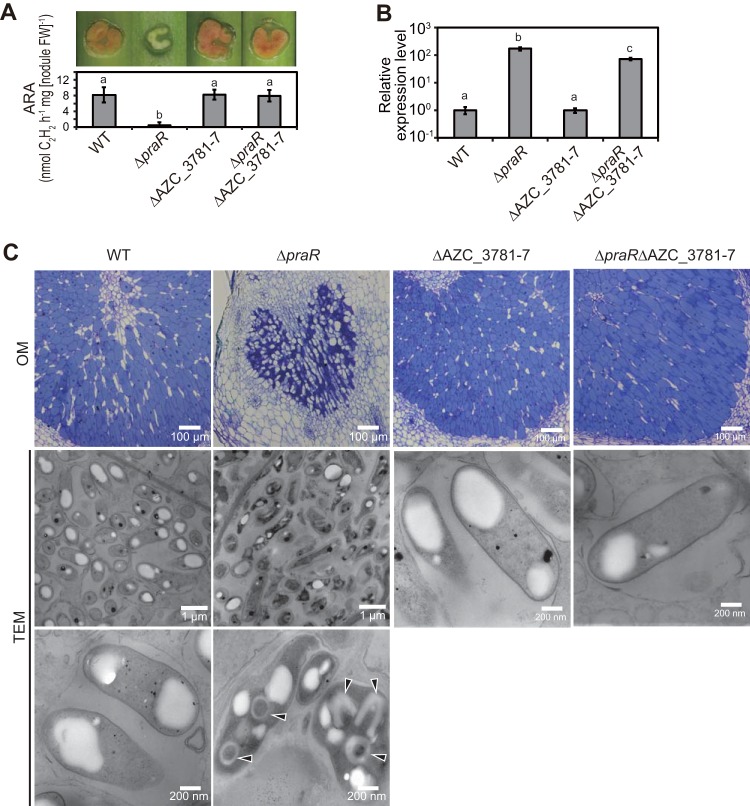
Contributions of the *reb* operon to R-body formation in stem nodules. *S. rostrata* plants were inoculated with the WT or Δ*praR*, ΔAZC_3781-7, or Δ*praR* ΔAZC_3781-7 mutant strains and grown at 30°C. Stem nodules were analyzed at 14 days postinoculation (dpi). (A) Hand-cut images (upper) and acetylene reduction activities (ARAs) reflecting nitrogen-fixing activities (lower) of stem nodules. Values are presented as means ± standard deviations from five separate plants. Different letters indicate significant differences (*P* < 0.05, Tukey-Kramer). (B) Quantitative reverse transcription-PCR (RT-PCR) analyses of the *reb* operon in stem nodules. Expression levels of the *reb* operon were normalized to 16S rRNA levels, expressed relative to corresponding data from the WT strain, and are presented as means ± standard deviations from three separate plants. Different letters indicate significant differences (*P* < 0.05, Tukey-Kramer). (C) Optical microscopic (OM) observations of infected host cells in stem nodules and transmission electron microscopic (TEM) observations of bacterial cells in host cells. Arrowheads indicate R bodies.

To identify roles of the *reb* operon in R-body formation, we generated a *praR* deletion (Δ*praR*) mutant, a deletion mutant with deletion of a region from AZC_3781 to AZC_3787 (ΔAZC_3781-7), and a Δ*praR* ΔAZC_3781-7 double mutant and observed phenotypes of stem nodules at 14 days postinoculation (dpi) with these mutant and wild-type (WT) bacteria. The nitrogen-fixation-defective (Fix^−^) phenotype of the stem nodules carrying bacteria with the *praR* deletion was suppressed by the second deletion in the AZC_3781-7 region (Fig. 2A), as observed previously (8), whereas the AZC_3781-7 deletion did not affect *reb* operon expression levels (Fig. 2B). Transmission electron microscopy (TEM) observations showed that R bodies were produced in many Δ*praR* bacterial cells in shrunken host cells, and many R-body-containing bacterial cells had collapsed appearances (Fig. 2C). R bodies were not observed in stem nodules harboring the double mutant (Fig. 2C), suggesting that genes that are essential for R-body formation are located in the region from AZC_3781 to AZC_3787.

**FIG 3:**
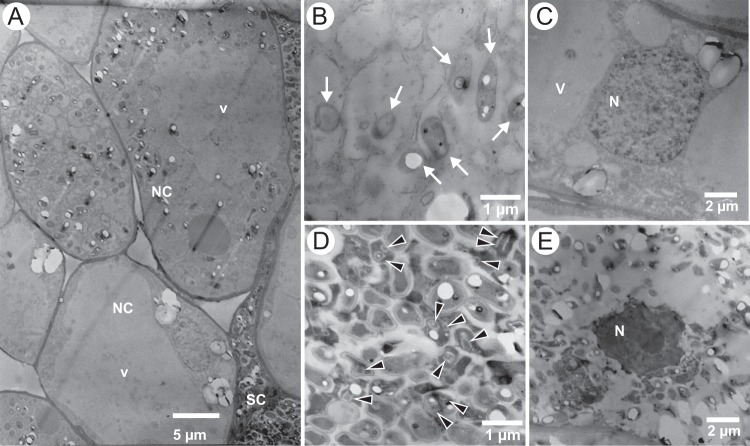
TEM observations of stem nodules harboring the Δ*praR* mutant at 7 dpi. (A) Normally shaped host cells (NC) with expanding vacuoles (v) and shrunken host cells (SC). (B and C) Bacterial cells (B) and host nuclei (C) in normally shaped cells. (D and E) Bacterial cells (D) and nuclei (E) in shrunken host cells. Arrows in panel B indicate bacterial cells, and arrowheads in panel D indicate R bodies. v, vacuole; N, nuclei.

To observe the dynamics of host-bacterium interactions in single nodules harboring the Δ*praR* mutant, pathogenic roles of R bodies were observed at an early stage of nodule development (7 dpi). Some normal-shape host cells contained bacterial cells that lacked R bodies (Fig. 3A and B), and nuclei in these normal-shape host cells were intact (Fig. 3C). In contrast, R bodies were observed in bacteria within shrunken host cells (Fig. 3A and D), in which nuclei were collapsed (Fig. 3E), indicating that R-body production is associated with host cell death in the nodules.

Requirements of the non-*reb*-homologous genes AZC_3784, AZC_3785, and AZC_3787 and the *reb*-homologous genes *reb*_*AZC1*_, *reb*_*AZC2*_, *reb*_*AZC3*_, and *reb*_*AZC4*_ for R-body production were determined in Δ*praR* mutant derivatives with the deletions of these genes. In these experiments, AZC_3784 was essential for R-body formation and AZC_3785 and AZC_3787 were not (see Fig. S3 in the supplemental material). In addition, both *reb*_*AZC3*_ and *reb*_*AZC4*_ and either *reb*_*AZC1*_ or *reb*_*AZC2*_ were essential for R-body production (see Fig. S4 in the supplemental material).

10.1128/mBio.00715-17.3 FIG S3  Contributions of AZC_3784, AZC_3785, and AZC_3787 genes to R-body formation. *S. rostrata* plants were inoculated with the indicated strains and were grown at 30°C and stem nodules were then analyzed at 14 dpi. (A) Hand-cut images (upper) and ARAs (lower) of stem nodules during symbiosis with the indicated strains. Data are presented as means ± standard deviations of five separate plants. Different letters indicate significant differences (*P* < 0.05, Tukey-Kramer). (B) Quantitative RT-PCR analysis of the *reb* operon in stem nodules. Expression levels of the *reb* operon were normalized to 16S rRNA levels and are presented as means ± standard deviations from three separate plants relative to corresponding transcripts from the WT strain. Different letters indicate significant differences (*P* < 0.05, Tukey-Kramer). (C) Optical microscopic (OM) observations of infected host cells in stem nodules and transmission electron microscopic (TEM) observations of residing bacterial cells. Arrowheads indicate R bodies. Deletions of AZC_3785 and AZC_3787 did not suppress the defective nitrogen-fixing phenotype of stem nodules that lacked *praR*, whereas deletion of AZC_3784 partially restored nitrogen fixation, as indicated by slight red color of the inner region of nodules harboring the Δ*praR* ΔAZC_3784 mutant. Expression levels of the *reb* operon were not affected by the AZC_3784 deletion, suggesting that partial suppression of the defective nitrogen-fixing phenotype of nodules was not caused by low expression of the *reb* operon. Host cells in nodules harboring the Δ*praR* ΔAZC_3784 mutant were oval or elongated, and shrunken host cells were not observed. Bacterial cell densities of the Δ*praR* ΔAZC_3784 mutant in host cells were not as high as for the WT strain. R bodies were not observed in Δ*praR* ΔAZC_3784 mutant bacteria, suggesting that the AZC_3784 gene is essential for R-body formation. R bodies were observed in Δ*praR* ΔAZC_3785 and Δ*praR* ΔAZC_3787 mutant bacteria, suggesting that the AZC_3785 and AZC_3787 genes are not essential for R-body formation. Download FIG S3, PDF file, 1.2 MB. Copyright © 2017 Matsuoka et al. 2017 Matsuoka et al. This content is distributed under the terms of the Creative Commons Attribution 4.0 International license.

10.1128/mBio.00715-17.4 FIG S4  Contributions of *reb*_*AZC1*_, *reb*_*AZC2*_, *reb*_*AZC3*_, and *reb*_*AZC4*_ genes to R-body formation. *S. rostrata* plants were inoculated with the indicated strains and were grown at 30°C. Stem nodules were analyzed at 14 dpi. (A) Hand-cut images (upper) and ARAs (lower) of stem nodules. Values are presented as means ± standard deviations from five separate plants. Different letters indicate significant differences (*P* < 0.05, Tukey-Kramer). (B) Quantitative RT-PCR analysis of the *reb* operon in stem nodules. Expression levels of the *reb* operon were normalized to 16S rRNA levels. Values are presented relative to respective WT expression data as means ± standard deviations from three separate plants. Different letters indicate significant differences (*P* < 0.05, Tukey-Kramer). (C) OM observations of infected host cells in stem nodules and TEM observations of the bacterial cells in host cells. Arrowheads indicate R bodies. Stem nodules that harbored Δ*praR* Δ*reb*_*AZC1*_ and Δ*praR* Δ*reb*_*AZC2*_ showed defective nitrogen-fixing phenotypes, and R bodies were observed in the residing bacteria. The Δ*praR* Δ*reb*_*AZC3*_, Δ*praR* Δ*reb*_*AZC4*_, Δ*praR* Δ*reb*_*AZC1*_ Δ*reb*_*AZC2*_, and Δ*praR* Δ*reb*_*AZC3*_ Δ*reb*_*AZC4*_ mutants formed normal stem nodules, and R bodies were not observed. Expression levels of the *reb* operon remained high in normal nodules, eliminating the possibility that the absence of R bodies reflected repression of *reb* operon expression. These results suggest that *reb*_*AZC3*_ and *reb*_*AZC4*_ and either *reb*_*AZC1*_ or *reb*_*AZC2*_ are essential for R-body production. Download FIG S4, PDF file, 1.1 MB. Copyright © 2017 Matsuoka et al. 2017 Matsuoka et al. This content is distributed under the terms of the Creative Commons Attribution 4.0 International license.

**FIG 4:**
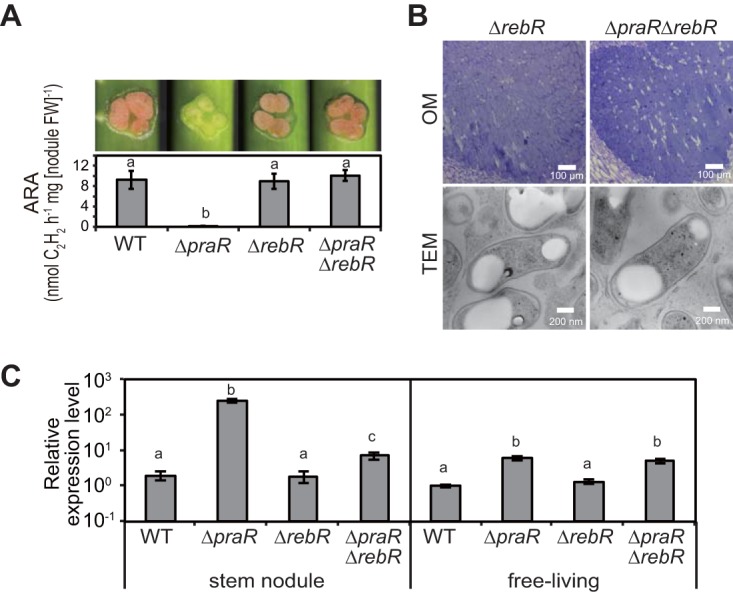
Phenotypes of *praR* and/or *rebR* mutants. *S. rostrata* plants were inoculated with the WT or Δ*praR*, Δ*rebR*, or Δ*praR* Δ*rebR* mutant strains and grown at 30°C, and stem nodules were analyzed at 14 dpi. (A) Hand-cut images (upper) and ARAs (lower) of stem nodules. Values are presented as means ± standard deviations from five separate plants. Different letters indicate significant differences (*P* < 0.05, Tukey-Kramer). (B) OM observations of infected host cells in stem nodules and TEM observations of harbored bacterial cells. (C) Relative expression levels of the *reb* operon in stem nodules and free-living cultures. Total RNAs were isolated from bacteria residing in stem nodules and from bacterial cultures after growth to an OD_600_ of approximately 1.0 at 38°C. Expression levels of the *reb* operon were estimated using quantitative RT-PCR and were normalized to 16S rRNA. Data are presented as means ± standard deviations of three replicate cultures and plants and are expressed relative to mRNA levels in free-living WT cultures. Statistical analyses were carried out for stem nodules and free-living cultures, respectively. Different letters indicate significant differences (*P* < 0.05; Tukey-Kramer).

In investigations of the contributions of RebR to nodule formation, deletion of *rebR* from the WT strain did not alter the phenotypic expression of stem nodules, but abolished the Fix^−^ phenotype of nodules harboring the Δ*praR* mutant so that the Δ*praR* Δ*rebR* mutant produced the Fix^+^ phenotype (Fig. 4A). R bodies were not observed in the Δ*praR* Δ*rebR* cells in the stem nodules (Fig. 4B). However, in symbiotic stem nodules, *reb* operon expression in the Δ*praR* mutant was more than 10-fold higher than that in the Δ*praR* Δ*rebR* mutant and was about 2 orders of magnitude higher than that in the WT strain, whereas levels of *reb* operon expression were similar in the WT and Δ*rebR* mutant strains (Fig. 4C). In contrast, in the free-living state, *reb* operon expression in the Δ*praR* mutant was similar to that in the Δ*praR* Δ*rebR* mutant. These results indicate that although PraR predominantly represses the expression of the *reb* operon, RebR acts an activator of the operon under conditions of symbiosis.

### PraR and RebR directly control the *reb* operon.

Following a systematic evolution of ligands by exponential enrichment (SELEX) analysis, Frederix et al. (18) proposed that PraR of *R. leguminosarum* binds the consensus palindrome CAAC-N5-GTTG. In the present SELEX analysis using N-terminally His_6_-tagged PraR (His_6_-PraR) from *A. caulinodans*, the consensus PraR sequence of *A. caulinodans* was also CAAC-N5-GTTG (see Fig. S5A in the supplemental material). However, no sequence on the promoter region of the *reb* operon (*reb* promoter) matched this consensus sequence perfectly or with a base substitution, whereas four sequences matched with two or three substitutions, and these were examined as candidates for PraR binding sites. Subsequent electrophoretic mobility shift assays (EMSAs) revealed that His_6_-PraR binds strongly to a sequence designated PraR-bs-A and weakly to the sequences of PraR-bs-B and -C (Fig. S5B and C). Meanwhile, SELEX analysis using N-terminally His_6_-tagged RebR (His_6_-RebR) revealed that RebR potentially binds to a consensus palindrome, GT(A/G)(A/C)C-N4-G(T/G)(T/C)AC (Fig. S5D). A sequence (designated RebR-bs) matching this consensus palindrome was present on the *reb* promoter, and the EMSA revealed that His_6_-RebR binds to this sequence (Fig. S5E). The positions of PraR-bs-A, -B, and -C and RebR-bs on the *reb* promoter are shown in Fig. 1.

10.1128/mBio.00715-17.5 FIG S5  Determination of PraR- and RebR-binding sites on the *reb* promoter. (A) SELEX experiment with His_6_-PraR of *A. caulinodans*. A dsDNA library containing a 20-bp random sequence flanked by M13 forward and M13 reverse sequences was prepared by PCR using the primer pair M13F-M13R and the oligonucleotide 5′-TCGAGCTCGGTACCCGACCAGACTG-N20-GTATGTGCGTGGGGATCCTCTAGAG-3′ as a template. The dsDNA library (10 pmol) was incubated with His_6_-PraR (100 pmol) in 100 µl of SELEX buffer (20 mM Tris-HCl [pH 8.0], 150 mM NaCl, 2 mM MgCl_2_, 20 mM imidazole, 0.05% Tween 20) supplied with 1 mM DTT and 10 ng µl^−1^ poly(dI-dC) for 30 min at 26°C. Ni-charged magnetic beads (His Mag Sepharose Ni; GE Healthcare) were used to capture His_6_-tagged proteins. Specifically, samples were combined with 1 µl of the magnetic beads (corresponding to 20 µl of 5% medium slurry), which were equilibrated in advance using SELEX buffer and incubated for 30 min at 26°C with gentle mixing by rotation. The beads capturing Hig_6_-tagged proteins were washed 10 times with 500 µl of the SELEX buffer. After the final washing, the beads were suspended in 50 µl of the SELEX buffer and were incubated at 95°C for 5 min. The eluted dsDNA was diluted 100 times with 10 mM Tris-HCl (pH 8.0) and amplified by PCR using primer pair M13F-M13R for subsequent rounds of selection. After five SELEX rounds, PCR products were cloned into pUC18 (C. Yanisch-Perron, J. Vieira, and J. Messing, Gene 33:103–119, 1985) using the In-Fusion cloning kit (Clontech), and variable regions (A-N20-C) of 48 clones were sequenced. All sequences were aggctaCAACtctagGTTGtgc. This sequence was aligned with the consensus palindrome sequence proposed by Frederix et al. (M. Frederix, A. Edwards, C. McAnulla, J. A. Downie, Mol Microbiol 81:994–1007, 2011, https://doi.org.10.1111/j.1365-2958.2011.07738.x.). No *reb* promoter sequences matched perfectly or had only one base difference from this consensus sequence, whereas four sequences with two or three different nucleotides (candidates 1, 2, 3, and 4) were present and were considered candidate PraR binding sites. (B) EMSA using FITC-labeled dsDNA probes that contained these candidate sequences. Probes were prepared by PCR using the primer pair M13F-M13R_FITC and oligonucleotides with candidate sequences flanked by M13 forward and reverse sequences. His_6_-PraR bound strongly to the candidate 2 sequence and weakly to candidate 3 and 4 sequences. His_6_-PraR did not bind the candidate 1 sequence. Thus, we designated the candidate sequences 2, 3, and 4 as PraR binding sites A, B, and C, respectively. (C) EMSA using FITC-labeled dsDNA probes with point mutations. Probes were prepared by PCR using oligonucleotides of the indicated sequences flanked by M13-forward and M13-reverse sequences and verified that PraR specifically binds these three sites. (D) SELEX experiment with His_6_-RebR of *A. caulinodans*. PCR products were cloned into pUC18 after five SELEX rounds, and variable regions of 32 clones were sequenced. Six sequences were thus obtained, and alignments of these suggested that RebR binds the consensus palindrome GT(A/G)(A/C)C-N4-G(T/G)(T/C)AC. A sequence matching this consensus with one different base was present on the *reb* promoter, and we considered this as a candidate RebR binding site. (E) EMSA using FITC-labeled dsDNA probes that contained this candidate sequence or point-mutated sequences verified that RebR specifically binds this sequence. Download FIG S5, PDF file, 0.3 MB. Copyright © 2017 Matsuoka et al. 2017 Matsuoka et al. This content is distributed under the terms of the Creative Commons Attribution 4.0 International license.

**FIG 5:**
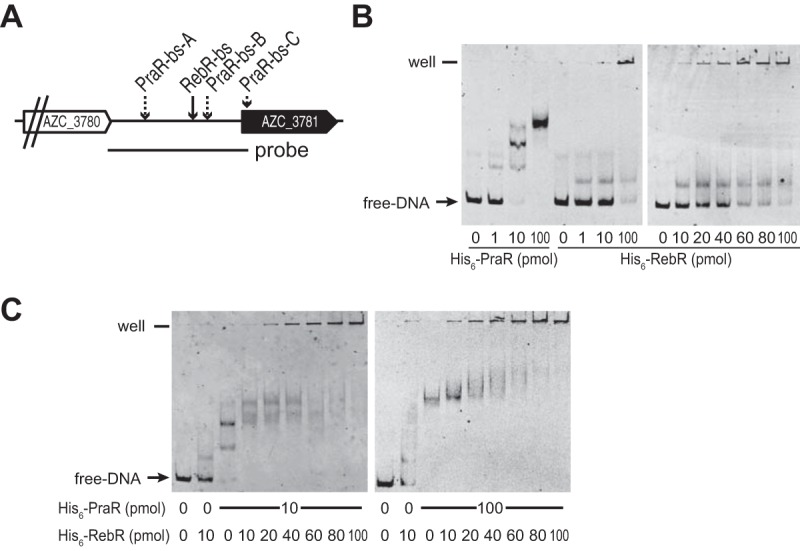
*In vitro* binding activities of His_6_-PraR and His_6_-RebR to the *reb* promoter. (A) The region of the FITC-labeled dsDNA probe used in EMSA analyses. (B) EMSA analysis of dsDNA probes with increasing levels of purified His_6_-PraR or His_6_-RebR. (C) EMSA analysis of dsDNA probes with both His_6_-PraR and His_6_-RebR.

To investigate whether PraR and RebR actually bind to the *reb* promoter, we carried out EMSAs using a double-stranded DNA (dsDNA) probe that covers the intergenic noncoding region upstream of the *reb* operon (*reb* promoter dsDNA probe) as shown in Fig. 5A. The molecular weight (MW) of the *reb* promoter dsDNA probe increased with the increasing quantity of His_6_-PraR, whereas addition of His_6_-RebR to the promoter probe increased not only the MW of the probe but also the amount of stacked probe in the wells of the gel in a concentration-dependent manner (Fig. 5B). Even in the presence of His_6_-PraR, the addition of His_6_-RebR to the promoter probe increased the MW of the probe (Fig. 5C), indicating that PraR does not interfere the binding of RebR to the RebR-bs.

**FIG 6:**
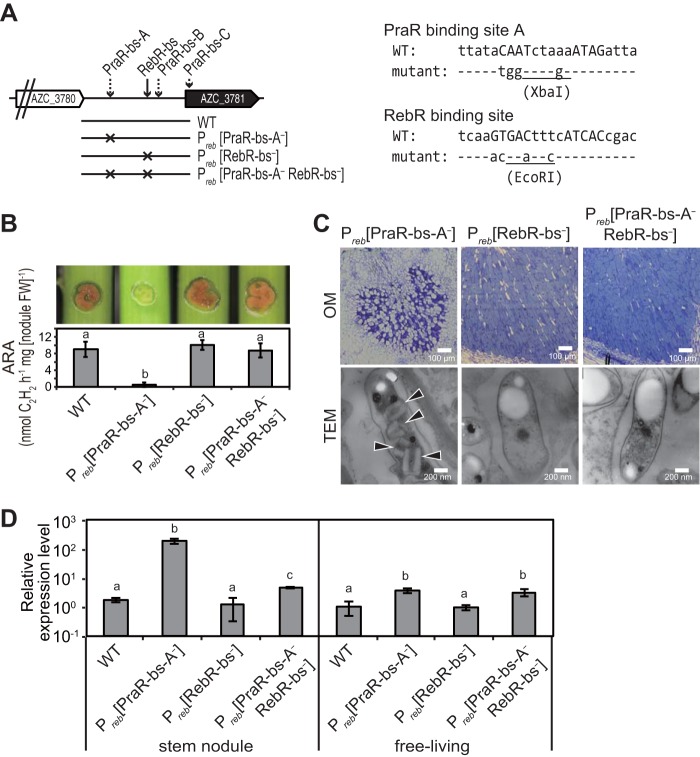
Phenotypes of PraR-bs-A and/or RebR-bs mutants. (A) Mutants with base substitutions in PraR-bs-A [P_*reb*_(PraR-bs-A^−^) mutant], RebR-bs [P_*reb*_(RebR-bs^−^) mutant], or both genes [P_*reb*_(PraR-bs-A^−^ RebR-bs^−^) double mutant] on the *reb* promoter. The sequences of the PraR binding site A and RebR binding site regions are shown in the right-hand side. The sequences corresponding to consensus palindromes are indicated in capital letters. Restriction endonuclease recognition sites (XbaI and EcoRI) were generated using the base substitutions. Mutants were inoculated into stems of *Sesbania* plants grown at 30°C, and stem nodules were analyzed at 14 dpi. (B) Hand-cut images (upper) and ARAs (lower) of the stem nodules formed by the indicated strains. Data are presented as means ± standard deviations from five separate plants. Different letters indicate significant differences (*P* < 0.05, Tukey-Kramer). (C) OM observations of infected host cells in stem nodules and TEM observations of residing symbiotic bacterial cells. Arrowheads indicate R bodies. (D) Relative expression levels of the *reb* operon in bacteria from stem nodules and free-living cultures. Total bacterial RNA was isolated from bacteria that were grown in stem nodules and from free-living cultures grown in the defined medium at 38°C to an OD_600_ of approximately 1.0. Expression levels of the *reb* operon were estimated using quantitative RT-PCR, normalized to 16S rRNA levels, and expressed relative to corresponding mRNA levels in free-living WT cultures and are presented as means ± standard deviations from three replicate cultures and plants. Statistical analyses were carried out for stem nodules and free-living cultures, respectively. Different letters indicate significant differences (*P* < 0.05, Tukey-Kramer).

To confirm the involvement of PraR and RebR in *reb* operon expression, the significance of the promoter sequences was assessed in stem nodules that were formed after inoculation with mutants carrying base substitutions of P_*reb*_(PraR-bs-A^−^) and P_*reb*_(RebR-bs^−^) mutants and after inoculation with the P_*reb*_(PraR-bs-A^−^ RebR-bs^−^) double mutant (Fig. 6A). Stem nodules harboring the P_*reb*_(PraR-bs-A^−^) mutant had the Fix^−^ phenotype, whereas those harboring the P_*reb*_(PraR-bs-A^−^ RebR-bs^−^) double mutant had restored nitrogen-fixing activity (Fig. 6B). Accordingly, R bodies were observed in P_*reb*_(PraR-bs-A^−^) mutants but not in P_*reb*_(PraR-bs-A^−^ RebR-bs^−^) double mutants in stem nodules (Fig. 6C). In further experiments, expression levels of the *reb* operon in P_*reb*_(PraR-bs-A^−^) mutants and P_*reb*_(PraR-bs-A^−^ RebR-bs^−^) double mutants were about 2 orders of magnitude and several times higher than those in the WT strain, respectively, whereas *reb* operon expression levels in the P_*reb*_(RebR-bs^−^) mutant were similar to those in the WT strain (Fig. 6D). The consistency of phenotypic expression between deletion mutants of *praR* and *rebR* (Δ*praR*, Δ*rebR*, and Δ*praR* Δ*rebR* mutants in Fig. 4) and corresponding promoter sequence mutants strongly indicates that PraR and RebR directly control the expression of the *reb* operon.

### Identification of environmental factors that abolish *reb* repression by PraR.

In the experiments described above, symbiotic R-body production was observed in Δ*praR* and P_*reb*_(PraR-bs-A^−^) mutants but was not present in the WT strain under symbiotic or free-living conditions, suggesting that R-body production is subjected to environmental conditions. Thus, we investigated environmental factors that induce RebR-dependent activation of the *reb* operon in the Δ*praR* mutant and then identified factors that attenuate PraR-dependent repression of the *reb* operon in WT cells grown under the identified favorable environmental conditions.

**FIG 7:**
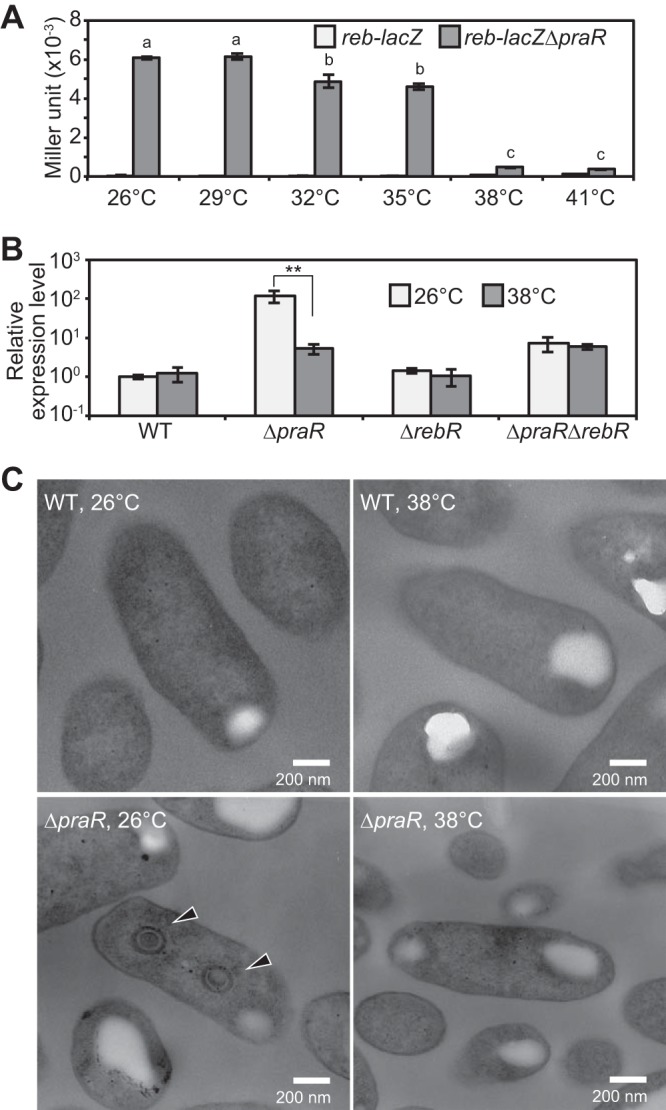
Effects of temperature on the expression of the *reb* operon and R-body formation in free-living *A. caulinodans* cells. (A) β-Galactosidase activities of *reb-lacZ* and *reb-lacZ* Δ*praR* strains. Bacterial cells were grown in the defined medium at the indicated temperatures to an OD_600_ of approximately 1.0, and β-galactosidase activities were measured. Data are presented as means ± standard deviations from three replicate cultures. Statistical analysis was carried out for the *reb-lacZ* Δ*praR* strain. Different letters indicate significant differences (*P* < 0.05, Tukey-Kramer). (B) Quantitative RT-PCR analysis of the *reb* operon in the WT and Δ*praR*, Δ*rebR*, and Δ*praR* Δ*rebR* mutant strains grown at 26 and 38°C. Data are expressed relative to those from the WT strain grown at 26°C and are presented as means ± standard deviations from three replicate cultures. Statistical analyses were carried out for each strain, respectively. Asterisks indicate significant difference (**, *P* < 0.01, Student’s *t* test). (C) TEM observations of free-living WT and Δ*praR* strains. Bacterial cells were collected from the same cultures that were used for the quantitative RT-PCR analyses described in panel B. Arrowheads indicate R bodies.

Initially, we constructed a *reb* operon-*lacZ* transcriptional fusion (*reb-lacZ*) on chromosomes of the WT and Δ*praR* strains, namely *reb-lacZ* and *reb-lacZ* Δ*praR* strains, respectively. During these manipulations, the *reb-lacZ* Δ*praR* strain expressed β-galactosidase in the free-living state at room temperature (around 26°C) but not at 38°C, suggesting that activation by RebR is temperature dependent. Accordingly, when *reb-lacZ* and *reb-lacZ* Δ*praR* strains were grown at various temperatures between 26 and 41°C, β-galactosidase activity was highly induced below 35°C in the *reb-lacZ* Δ*praR* strain, but not in the *reb-lacZ* strain (Fig. 7A). Moreover, expression levels of the *reb* operon were about 30-fold higher at 26°C than at 38°C in the Δ*praR* strain, whereas activation at 26°C was not observed in either the Δ*rebR* or Δ*praR* Δ*rebR* mutant (Fig. 7B). Under free-living conditions, R bodies were observed in up to 10% of Δ*praR* cells grown at 26°C, but not in those grown at 38°C (Fig. 7C). Similarly, Δ*praR* cells in stem nodules (symbiotic state) failed to produce R bodies when plants were grown at 38°C, and *reb* operon expression was lower than that in Δ*praR* cells under symbiotic conditions at 30°C (see Fig. S6 in the supplemental material). Taken together, these data indicate that activation of the *reb* operon by RebR is temperature dependent under both free-living and symbiotic conditions. However, binding of RebR to the *reb* promoter was not affected by temperature (see Fig. S7 in the supplemental material).

10.1128/mBio.00715-17.6 FIG S6  Effects of high temperature on the phenotypes of the stem nodules. *S. rostrata* plants were inoculated with WT and Δ*praR* strains and were grown at 30 or 38°C. Stem nodules were then analyzed at 14 dpi. (A) Hand-cut images (upper) and ARAs (lower) of the stem nodules. Data are presented as means ± standard deviations from five separate plants. Different letters indicate significant differences (*P* < 0.05, Tukey-Kramer). (B) OM observations of infected host cells in stem nodules and TEM observations of the bacterial cells in host cells. (C) Quantitative RT-PCR analysis of the *reb* operon in stem nodules. Expression levels of the *reb* operon were normalized to 16S rRNA levels and are expressed relative to WT values at 30°C. Values are presented as means ± standard deviations from three separate plants. Different letters indicate significant differences (*P* < 0.05, Tukey-Kramer). Download FIG S6, PDF file, 0.4 MB. Copyright © 2017 Matsuoka et al. 2017 Matsuoka et al. This content is distributed under the terms of the Creative Commons Attribution 4.0 International license.

10.1128/mBio.00715-17.7 FIG S7  Effects of temperature and 2OG on *in vitro* binding activities of His_6_-RebR to the *reb* promoter. (A) The region of the dsDNA probes. (B) Quantitative PCR analysis to investigate the effects of temperature on the *reb* promoter binding activities of His_6_-RebR. The dsDNA probes (1 pmol) corresponding to the *reb* promoter of WT and Preb(RebR-bs^−^) strains were incubated with purified His_6_-RebR (1, 10, or 100 pmol) in 50 µl of SELEX buffer supplied with 1 mM DTT and 10 ng µl^−1^ poly(dI-dC) for 1 h at 26 or 38°C. Samples were combined with 0.5 µl of Ni-charged magnetic beads (corresponding to 10 µl of 5% medium slurry of His Mag Sepharose Ni; GE Healthcare) and were incubated at 26 or 38°C for 1 h. Beads that captured His_6_-RebR were washed 10 times with 500 µl of SELEX buffer at 26 or 38°C. After the final wash, the beads were suspended with 50 µl of the SELEX buffer and incubated at 95°C for 5 min to recover dsDNA from His_6_-RebR. Recovered dsDNA contents in eluted solutions were measured by quantitative PCR. (C) EMSA analysis to investigate the effects of 2OG on the binding activities of His_6_-RebR to the *reb* promoter. An FITC-labeled dsDNA probe corresponding to the *reb* promoter was incubated with His_6_-RebR in the presence of 0 to 100 mM 2OG at 26°C. Download FIG S7, PDF file, 0.2 MB. Copyright © 2017 Matsuoka et al. 2017 Matsuoka et al. This content is distributed under the terms of the Creative Commons Attribution 4.0 International license.

**FIG 8:**
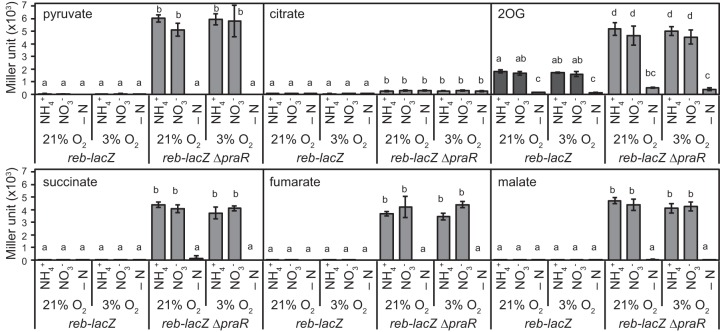
Effects of carbon and nitrogen sources and oxygen concentrations on the expression of the *reb* operon under free-living conditions. Mutant *reb-lacZ* and *reb-lacZ* Δ*praR* strains were grown in medium containing 50 mM pyruvate, citrate, 2-oxoglutarate (2OG), succinate, fumarate, or malate as carbon sources and 10 mM NH_4_^+^ or NO_3_^−^ as nitrogen sources or without a nitrogen source (−N) under aerobic (21% O_2_) or microaerobic (3% O_2_) conditions. Initial OD_600_ values of cultures were adjusted to 0.02 for pyruvate, succinate, fumarate, and malate media with NH_4_^+^ or NO_3_^−^ supplementation, 0.1 for supplementation with 2OG and NH_4_^+^ or NO_3_^−^, and 0.2 for supplementation with citrate medium and NH_4_^+^ or NO_3_^−^ or nitrogen-deficient media. After incubation at 26°C for 24 h, β-galactosidase activities were measured. Data are presented as means ± standard deviations from three replicate cultures. Different letters indicate significant differences (*P* < 0.05, Tukey-Kramer).

**FIG 9:**
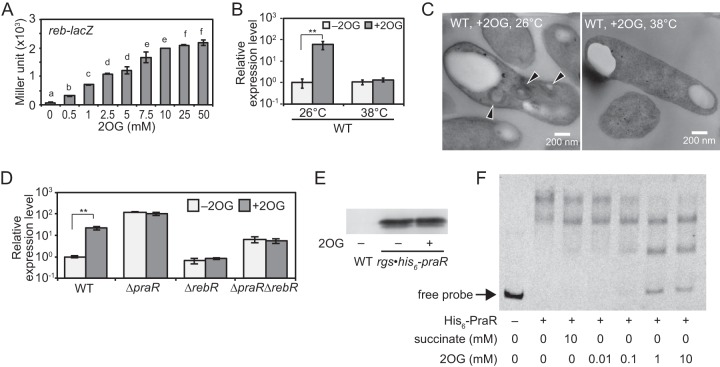
Effects of 2OG on the expression of the *reb* operon and R-body formation in bacteria under free-living conditions. (A) β-Galactosidase activities of the *reb-lacZ* strain in the presence of various 2OG concentrations. The *reb-lacZ* strain was grown in medium with the indicated concentrations of 2OG (0 to 50 mM) at 26°C for 24 h to an OD_600_ of approximately 1.0. Data are presented as means ± standard deviations from three replicate cultures. Different letters indicate significant differences (*P* < 0.05, Tukey-Kramer). (B) Quantitative RT-PCR analysis to investigate the effects of 2OG and temperature on the *reb* operon expression in the WT strain. Cultures of the WT strain were supplemented with 10 mM 2OG (+2OG) or no 2OG (−2OG) and were grown at 26 or 38°C. The data are expressed relative to those from the cultures under –2OG conditions at 26°C and are presented means ± standard deviations from three replicate cultures. Asterisks indicate significant difference (**, *P* < 0.01, Student’s *t* test). (C) TEM observations of the WT cells cultured with 2OG at 26 and 38°C. Bacterial cells were collected from the cultures that were used for the quantitative RT-PCR conducted in panel B. Arrowheads indicate R bodies. (D) Quantitative RT-PCR analysis of the *reb* operon in the WT, Δ*praR*, Δ*rebR*, and Δ*praR* Δ*rebR* strains. These strains were grown under +2OG or –2OG conditions at 26°C. The data are expressed relative to corresponding values in the WT strain under –2OG conditions at 26°C and are presented as means ± standard deviations from three replicate cultures. Asterisks indicate significant difference (**, *P* < 0.01, Student’s *t* test). (E) Western blotting with an anti-His_5_ antibody to investigate the effects of 2OG on RGS-His_6_-PraR expression. Whole-cell lysates from the *rgs-his_6_-praR* strain grown under +2OG or –2OG conditions and from the WT strain grown under –2OG conditions were electrophoresed. (F) EMSA analysis to investigate the effects of 2OG on binding activities of His_6_-PraR to the *reb* promoter. FITC-labeled dsDNA probe corresponding to the *reb* promoter was incubated with the purified His_6_-PraR in the presence of 2OG (0.01 to 10 mM) or succinate (10 mM).

In subsequent experiments, the effects of host-derived tricarboxylic acid (TCA) cycle, nitrogen, and oxygen metabolites on *reb* expression were investigated in *reb-lacZ* and *reb-lacZ* Δ*praR* strains. In the absence of *praR* (the *reb-lacZ* Δ*praR* strain), all organic acids except for citrate promoted *reb* expression under nitrogen-sufficient conditions at either 21 or 3% oxygen (Fig. 8). However, even in the presence of *praR* (*reb-lacZ* strain), *reb* expression was induced in medium containing 2-oxoglutarate (2OG) with sufficient nitrogen sources (Fig. 8). Moreover, induction was increased with 2OG concentrations even in the presence of succinate as a carbon source (Fig. 9A), suggesting that 2OG induces *reb* irrespective of its metabolism as a carbon source, although these effects of 2OG were observed at 26°C but not at 38°C (Fig. 9B). In agreement, TEM observations showed that R bodies were produced in WT cells grown in the presence of 2OG at 26°C but not at 38°C (Fig. 9C). Taken with the absence of a response to 2OG in Δ*rebR* mutant cells (Fig. 9D), these observations indicate that *rebR* is essential for 2OG-mediated induction of the *reb* operon. Moreover, the Δ*praR* mutants did not respond to 2OG, further suggesting that 2OG derepresses the *reb* operon by attenuating PraR-dependent repression. Western blotting of an *rgs-his_6_-praR* transformant (19) showed that expression levels of RGS-His_6_-PraR protein were not affected by 2OG (Fig. 9E). However, binding of PraR to the *reb* promoter dsDNA probe decreased with increasing 2OG concentration, whereas RebR binding was impervious to 2OG (Fig. 9F; Fig. S7). These observations strongly suggest that 2OG derepresses the *reb* operon directly by concentration-dependently inhibiting PraR binding to the *reb* promoter.

## DISCUSSION

In this study, we demonstrated that *reb*-driven pathogenicity is associated with R-body production by *A. caulinodans* and suggest that R-body production is a widespread trait among bacterial pathogens that carry *reb* operons. Although the *reb* operon is predominantly repressed by PraR, we identified biological and environmental factors that derepress *reb* gene expression and thus R-body production under free-living conditions.

R bodies are rolled up at neutral pH, but reportedly unroll to form needle-shaped structures at low pH (10). Recombinant R bodies from *Escherichia coli* act as pistons that puncture spheroplasts of *E. coli* at low pH (20). In paramecia, the role of R bodies in the killer trait follows release of R-body-containing bacteria from killer paramecia and ingestion by sensitive paramecia. Subsequently, internalized bacteria enter acidified food vacuoles, and R bodies are unrolled and penetrate the phagosomal membrane to deliver lethal toxins to the cytoplasm (9, 21). This scenario may also be applicable to interactions of *A. caulinodans* and *S. rostrata*, in which the peribacteroid space (microenvironment surrounding bacteroids) is progressively acidified during nodule morphogenesis (22), likely triggering the conformational change of R bodies into the needle-shaped structure that penetrates membranes.

The present series of mutant analyses showed that essential genes for R-body formation in *A. caulinodans* include both *reb*-homologous and non-*reb*-homologous genes. Although the roles of the proteins encoded by the *reb*-homologous genes remain poorly understood, they are likely to be components of R bodies (5). Among non-*reb*-homologous genes, AZC_3784 was found in the *reb* operon and was also essential to R-body formation. In *C. taeniospiralis*, RebC is encoded by a non-*reb*-homologous gene that may be involved in the assembly of R bodies (5). Similarly, the AZC_3784 protein is not a component of R bodies but is likely involved in their assembly, although it may lack homology to *rebC* from *C. taeniospiralis*. Taken together, these observations warrant further compositional analyses of R bodies and investigations of the molecular mechanisms of R-body formation.

R bodies were frequently produced by Δ*praR* mutants in the symbiotic state, but were observed in fewer than 10% of free-living cells, even at the optimum temperature (26°C) for *reb* operon expression. These results suggest that R-body formation is more strongly regulated (suppressed) in the free-living state—probably at the translation level or at the R-body assembly level—and that *A. caulinodan* R bodies play more important roles in the symbiotic state.

Although R-body formation was not observed in WT bacterial cells in the symbiotic state, the present environmental factors that derepress the *praR* regulatory system have significant implications for the understanding of *reb* operon evolution during microsymbiosis of *A. caulinodan* with *S. rostrata*. It is widely accepted that virulence genes are regulated by temperature in many pathogenic bacteria and are upregulated in mammalian bacterial pathogens at around host body temperature (37°C) (23, 24). In contrast, most plant-pathogenic bacteria express virulence genes at ambient temperatures that are generally lower than their optimal growth temperatures (25). For example, *Agrobacterium tumefaciens* mediates the formation of crown galls at temperatures below 32°C, and the VirA/VirG two-component system regulates the expression of virulence genes according to temperature (26, 27). In agreement, the *reb* operon was expressed in the Δ*praR* mutant of *A. caulinodans* at temperatures below 35°C and within the optimal range for the growth of the host plant (around 30°C). Moreover, because regulation by PraR was derepressed by 2OG in the present free-living WT cells at 26°C, the *reb* operon may also be induced during symbiosis in host nodules, wherein the 2OG is accumulated in the host plant cells, although we have not found the conditions under which 2OG actually accumulates in the host plant cells. Plant 2OG levels reflect cellular C/N status and may play a signaling role in the coordination of C and N metabolism (28). Alterations in the activities of nitrogen fixation by bacteria and ammonia assimilation by plant cells may lead to 2OG accumulation in host cells. To elucidate the roles of *reb* operon in the symbiotic state, we need to conduct more investigations to estimate the conditions wherein 2OG is accumulated in the host plant cells.

Although PraR homologues are widespread among *Alphaproteobacteria* (8), the roles of PraR have not been well characterized. In particular, the *praR* homologue *phrR* was originally identified in the acid-tolerant rhizobium *Sinorhizobium medicae* WSM419 as a gene that is induced at low pH (29). However, *praR* expression is not pH sensitive in *A. caulinodans* and *Rhizobium leguminosarum* (8, 18). Moreover, *R. leguminosarum* PraR directly represses the expression of the quorum-sensing genes *rhiR* and *raiR* and the biofilm formation genes *rapA2*, *rapB*, and *rapC* (18, 30), whereas the homologous genes in *A. caulinodans* are not controlled by PraR (8). Hence, although PraR homologues are widely distributed, the roles of PraR have diversified during the evolution of *Alphaproteobacteria*. The ubiquity of chemical and environmental factors that regulate *praR* expression in the *Alphaproteobacteria*, such as the effects of *A. caulinodans* factors, also requires investigation in the context of the evolution of *praR* regulatory systems.

RebR belongs to a novel subfamily of the Crp-Fnr superfamily, and all Crp-Fnr members carry putative DNA-binding helix-turn-helix domains on their C terminus and ligand-binding domains on their N terminus (17). Various intracellular and exogenous signals activate Crp-Fnr members via their ligand-binding domains, including 2OG and temperature (17). In *A. caulinodans*, however, binding activity of RebR to the *reb* operon was not affected by 2OG and temperature, indicating that in addition to 2OG and temperature, as yet unidentified factors are involved in the activation of *reb* operon expression via RebR.

Herein, we demonstrated the roles of R bodies in the pathogenicity of bacteria that harbor the *reb* operon, although the ensuing roles in nodule symbiosis and the related evolutionary implications remain uncharacterized. Because bacterial genomes are plastic, endosymbionts may become pathogenic after acquiring the *reb* operon if they do not suppress its expression. Although we did not determine whether R bodies threaten biodiversity or ecosystems, this possibility may require solutions in the future. Unlike obligate endosymbionts of paramecia, *A. caulinodans* can be cultured *in vitro* and genetic manipulation techniques have been established in this bacterium, warranting further use of *A. caulinodans* as a model for studies of R-body/*reb* genes.

## MATERIALS AND METHODS

### Bacterial strains and culture conditions.

The bacterial strains used in this study are listed in Table S1 in the supplemental material. *A. caulinodans* strains were grown in tryptone-yeast extract (TY) medium (31) or in basal defined (BD) medium containing 50 mM disodium succinate, 10 mM NH_4_Cl, 10 mM potassium phosphate (pH 7.0), 100 mg liter^−1^ MgSO_4_⋅7H_2_O, 50 mg liter^−1^ NaCl, 40 mg liter^−1^ CaCl_2_⋅2H_2_O, 5.4 mg liter^−1^ FeCl_3_⋅6H_2_O, 5 mg liter^−1^ Na_2_MoO_4_⋅2H_2_O, 2 mg liter^−1^ biotin, 4 mg liter^−1^ nicotinic acid, and 4 mg liter^−1^ pantothenic acid. To vary the carbon and nitrogen contents of BD medium, disodium succinate was replaced with sodium pyruvate, trisodium citrate, disodium 2-oxoglutarate, disodium fumarate, or disodium l-malate, and NH_4_Cl was replaced with KNO_3_ or omitted. In some experiments, BD medium was further supplemented with disodium 2OG. To grow *A. caulinodans* strains under aerobic conditions, test tubes containing medium were sealed with butyl rubber septums, and the contained air was replaced with N_2_ gas with 3% O_2_. Before inoculation into BD medium, bacterial cells were cultured overnight in TY medium and were washed twice in 10 mM potassium phosphate buffer (pH 7.0). Unless otherwise noted, initial optical density at 600 nm (OD_600_) values of cultures were adjusted to 0.1 or 0.02 for growth at 26 or 38°C, respectively, and OD_600_ values were approximately 1.0 after 24 h of incubation.

10.1128/mBio.00715-17.8 TABLE S1  Bacterial strains and plasmids used in this study. Download TABLE S1, DOCX file, 0.1 MB. Copyright © 2017 Matsuoka et al. 2017 Matsuoka et al. This content is distributed under the terms of the Creative Commons Attribution 4.0 International license.

### Construction of deletion and substitution mutants.

The plasmids and primers used for strain construction are listed in Tables S1 and S2 in the supplemental material, respectively.

To construct *A. caulinodans* deletion mutants of AZC_3784, AZC_3785, AZC_3787, and *rebR* genes, two DNA fragments containing upstream and downstream regions of each gene were amplified from the WT genomic DNA by PCR using appropriate primer pairs and were then directionally cloned into a suicide vector, pK18*mobsacB* (32), using the In-Fusion cloning kit (Clontech, Mountain View, CA). The linearization of pK18*mobsacB* was performed by inverse PCR using the PrimeSTAR Max (TaKaRa-Bio, Shiga, Japan) with primer pair Tp73-Tp74. The resulting plasmids were conjugated into the WT or Δ*praR* (8) strains via *E. coli* S17-1(λpir) (33), and gene deletions were introduced by allelic exchange.

10.1128/mBio.00715-17.9 TABLE S2  Primers used in this study. Download TABLE S2, DOCX file, 0.1 MB. Copyright © 2017 Matsuoka et al. 2017 Matsuoka et al. This content is distributed under the terms of the Creative Commons Attribution 4.0 International license.

To construct deletion mutants of the *reb*_*AZC1*_, *reb*_*AZC2*_, *reb*_*AZC3*_, and *reb*_*AZC4*_ genes, a series of plasmids were constructed as follows. DNA fragments containing the WT AZC_3781-7 region with its upstream and downstream regions were amplified by PCR and cloned into the linearized pK18*mobsacB*. Genes on plasmids containing the WT region were deleted by inverse PCR using the PrimeSTAR Mutagenesis Basal kit (TaKaRa-Bio). To introduce double deletions, second inverse PCRs were conducted using plasmids harboring single mutations. Constructed plasmids were conjugated into the Δ*praR* ΔAZC_3781-7 mutant, and deletion mutants were obtained after allelic exchange.

To construct mutants with base substitutions in PraR-bs-A and/or RebR-bs, a series of plasmids were constructed as follows. A DNA fragment containing the WT *reb* promoter region was amplified by PCR and cloned into the linearized pK18*mobsacB*. An XbaI site was generated within the PraR-bs-A on the plasmid containing the *reb* operon by inverse PCR using the PrimeSTAR Mutagenesis Basal kit. Similarly, an EcoRI site was generated within the RebR-bs on the plasmid containing the *reb* promoter. The resulting plasmids were conjugated into the WT strain, and mutants were obtained after allelic exchange.

To construct strains that express the *reb-lacZ* fusion gene, two fragments containing *rebR* and AZC_3789 and a *lacZ* fragment were amplified by PCR from the WT genomic DNA and the plasmid pTA-MTL (34), respectively. Fragments were then cloned into the linearized pK18*mobsacB* in the direction of the *rebR*, *lacZ*, and AZC_3789 fragments using the In-Fusion cloning kit. The plasmid containing *reb-lacZ* was conjugated into the WT and Δ*praR* strains, and strains with *lacZ* at the position immediately downstream of the *rebR* open reading frame (ORF) were obtained after allelic exchange.

### Plant growth and bacterial inoculation for nodule formation.

*S. rostrata* stems were inoculated with *A. caulinodans* strains as described previously (19) and were then grown at 30 or 38°C under a 24-h light regimen. Acetylene reduction activities (ARAs) of stem nodules were assayed as described previously (19).

### Optical microscopy observation.

Stem nodules were longitudinally cut into three pieces. The middle pieces of each sample were chemically fixed with 4% paraformaldehyde and 2% glutaraldehyde, dehydrated through a graded ethanol series, and then embedded in Technovit 7100 (Heraeus Kulzer). The embedded samples were sliced into 5-µm sections, stained with 0.05% toluidine blue O, and then observed using a bright-field microscope (DMLB; Leica).

### TEM observation.

Bacterial cells were collected from culture media by centrifugation. Stem nodules were cut into small pieces. These samples were chemically fixed as described above, postfixed with 2% OsO_4_, dehydrated through a graded ethanol series, and finally embedded in Spurr low-viscosity embedding medium (Polysciences, Warrington, PA). Embedded samples were then sliced into ultrathin (about 70-nm) sections, stained with uranyl acetate followed by lead citrate, and examined using a JEM-1010 electron microscope (JEOL, Tokyo, Japan) at an accelerating voltage of 100 kV.

### β-Galactosidase assay.

β-Galactosidase activity was measured according to a previously reported method (35) with some modifications as follows. Fifty microliters of bacterial cultures was mixed with 450 µl of Z buffer (60 mM Na_2_HPO_4_, 40 mM NaH_2_PO_4_, 10 mM KCl, 1 mM MgSO_4_) supplied with 50 mM 2-mercaptoethanol and 0.001% sodium dodecyl sulfate (SDS) and 50 µl of chloroform. The mixture was vortexed for 30 s, and 50 µl of *o*-nitrophenyl-β-d-galactoside (ONPG [4 mg ml^−1^ in Z buffer]) was added. After incubation at 25°C, reactions were stopped by adding 250 µl of 1 M Na_2_CO_3_. Mixtures were centrifuged, and 200 µl of the supernatants was transferred to 96-well clear plates. Subsequently, 200 µl of the bacterial cultures was transferred to 96-well clear plates. The absorbance of the supernatant at 415 and 540 nm and optical density at 595 nm (OD_595_) of the cultures were measured using a microplate reader (680 XR; Bio-Rad, Hercules, CA).

### Total RNA extraction and quantitative RT-PCR.

Total RNA was isolated from bacterial cultures and stem nodules, and cDNA was synthesized according to previously described methods (19). Quantitative PCR was performed with a LightCycler system (Roche, Basel, Switzerland) using the QuantiTect SYBR green PCR kit (Qiagen, Hilden, Germany) with primer pairs Acp326-Acp714 for the *reb* operon and Tp35-Tp36 for 16S rNRA. Standard curves were generated using genomic DNA that was isolated from the WT strain using the NucleoSpin tissue kits (Macherey-Nagel, Düren, Germany). To determine expression levels of the *reb* operon, copy numbers of *reb* operon transcripts were normalized to those of 16S rRNA.

### Purification of His_6_-tagged PraR and RebR.

The *praR* ORF was amplified by PCR from the WT genomic DNA using primer pair Acp375-Acp161 and was then cloned into BamHI and XbaI restriction sites of the pCold I vector (TaKaRa-Bio, Shiga, Japan). The resulting plasmid was designated pTAC99. The *rebR* ORF was amplified by PCR from WT genomic DNA using primer pair Acp669-Acp670 and cloned into pCold I using the In-Fusion cloning kit (Clontech, Mountain View, CA). The linearization of pCold I was performed by inverse PCR using the PrimeSTAR Max (TaKaRa-Bio) with primer pair Tp78-Tp79. The resulting plasmid was designated pTAC133. pTAC99 and pTAC133 were transformed into *E. coli* BLR(DE3) (Novagen, Darmstadt, Germany). Subsequently, His_6_-PraR and His_6_-RebR were extracted and purified using Ni^+^-charged magnetic beads (His Mag Sepharose Ni; GE Healthcare, Little Chalfont, United Kingdom). Eluted proteins were then concentrated using Vivaspin 500 kits (molecular weight cutoff [MWCO] of 5,000; Sartorius, Göttingen, Germany) and were mixed with 5 volumes of 1.2× storage buffer (24 mM Tris-HCl [pH 8.0], 120 mM NaCl, 1.2 mM dithiothreitol [DTT], 60% glycerol).

### EMSA of the *reb* promoter.

Fluorescein isothiocyanate (FITC)-labeled dsDNA probes corresponding to the *reb* promoter region were prepared as follows. Initially, the *reb* promoter region was amplified by PCR using primer pair Acp646-Acp702 and genomic DNA from WT or derivative strains. Acp702 has an M13 reverse sequence at the 5′ end. PCR products from each strain were purified using the MinElute PCR purification kit (Qiagen) and were used as the templates for the second round of PCR using primer pair Acp646-M13R_FITC. Finally, FITC-labeled PCR products were purified. EMSAs were then performed by incubating 1-pmol aliquots of FITC-labeled dsDNA probes with various amounts of His_6_-PraR and/or His_6_-RebR in 20 µl of EMSA buffer [20 mM Tris-HCl (pH 8.0), 1 mM EDTA, 60 mM NaCl, 5 mM MgCl_2_, 1 mM DTT, 10 ng µl^−1^ poly(dI-dC), 4% glycerol, 0.05% Tween 20] for 30 min at 26°C. Binding reaction mixtures were then electrophoresed in 4% native polyacrylamide gels containing 2.5% glycerol in 0.5× Tris-borate-EDTA (TBE) buffer, and FITC-labeled DNAs were detected using the LAS3000 system (Fuji Film, Tokyo, Japan).

### Western blotting analyses of RGS-His_6_-PraR.

Bacterial cells were collected from cultures by centrifugation, sonicated in phosphate-buffered saline (150 mM NaCl, 10 mM Na_2_HPO_4_, 20 mM NaH_2_PO_4_ [pH 7.4]), and then fractionated by SDS-PAGE using 14% polyacrylamide gels. Fractionated proteins were electroblotted onto polyvinylidene difluoride (PVDF) membranes, incubated with mouse anti-His_5_ antibody (Qiagen), and detected using horseradish peroxidase (HRP)-conjugated sheep anti-mouse antibodies (GE Healthcare) and the EzWestLumi Plus reagents (Atto, Tokyo, Japan).
