# Supplementary figures and images for: Stringent Expression Control of Pathogenic R-body Production in Legume Symbiont Azorhizobium caulinodans
Source: mBio. 2017 Jul 25;8(4):e00715-17. doi: 10.1128/mBio.00715-17 (PMC5527310; doi:10.1128/mBio.00715-17)

**A**

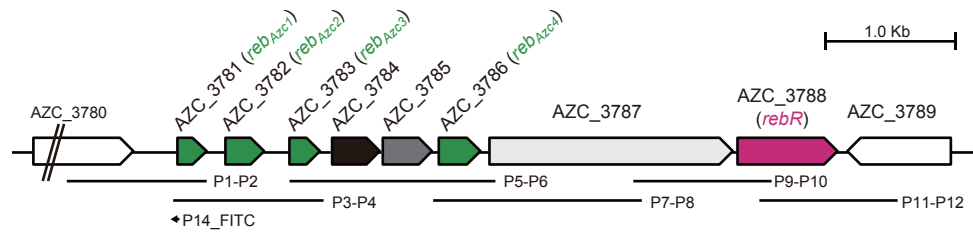

**B**

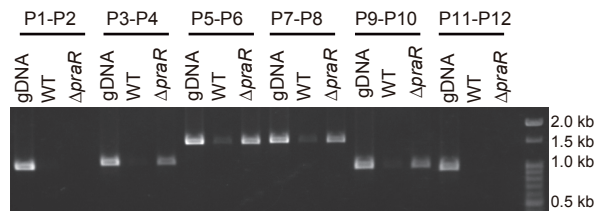

**C**

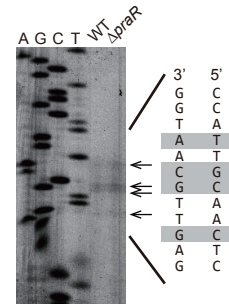

Supplement: FIG S1 [file mbo004173406sf1.pdf]

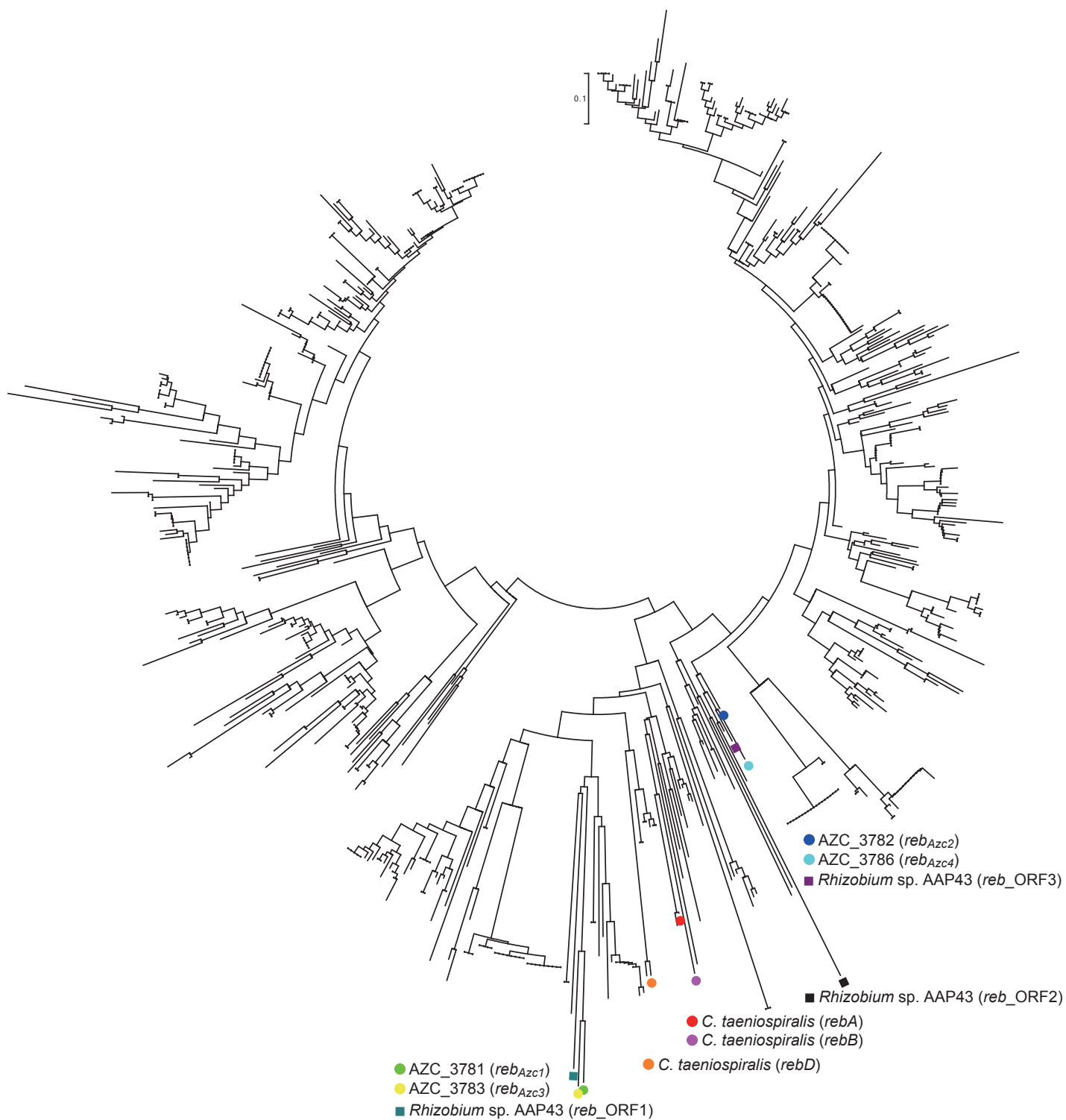

Supplement: FIG S2 [file mbo004173406sf2.pdf]

**A**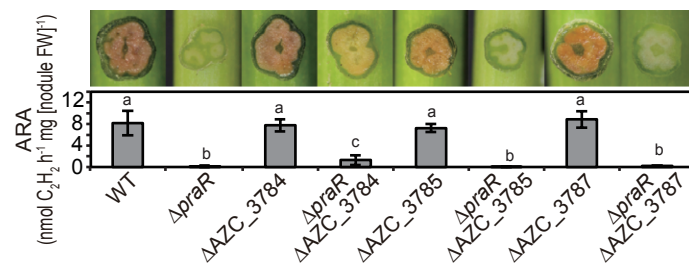**B**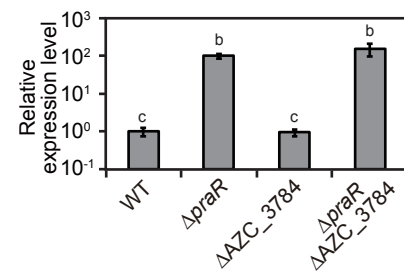**C**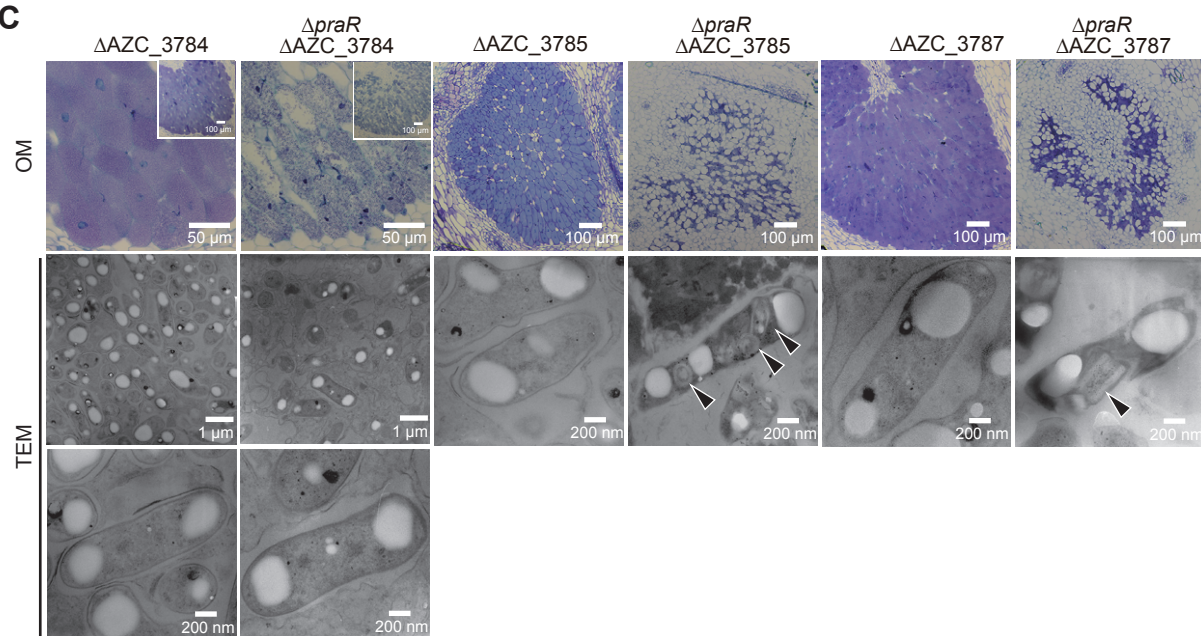

Supplement: FIG S3 [file mbo004173406sf3.pdf]

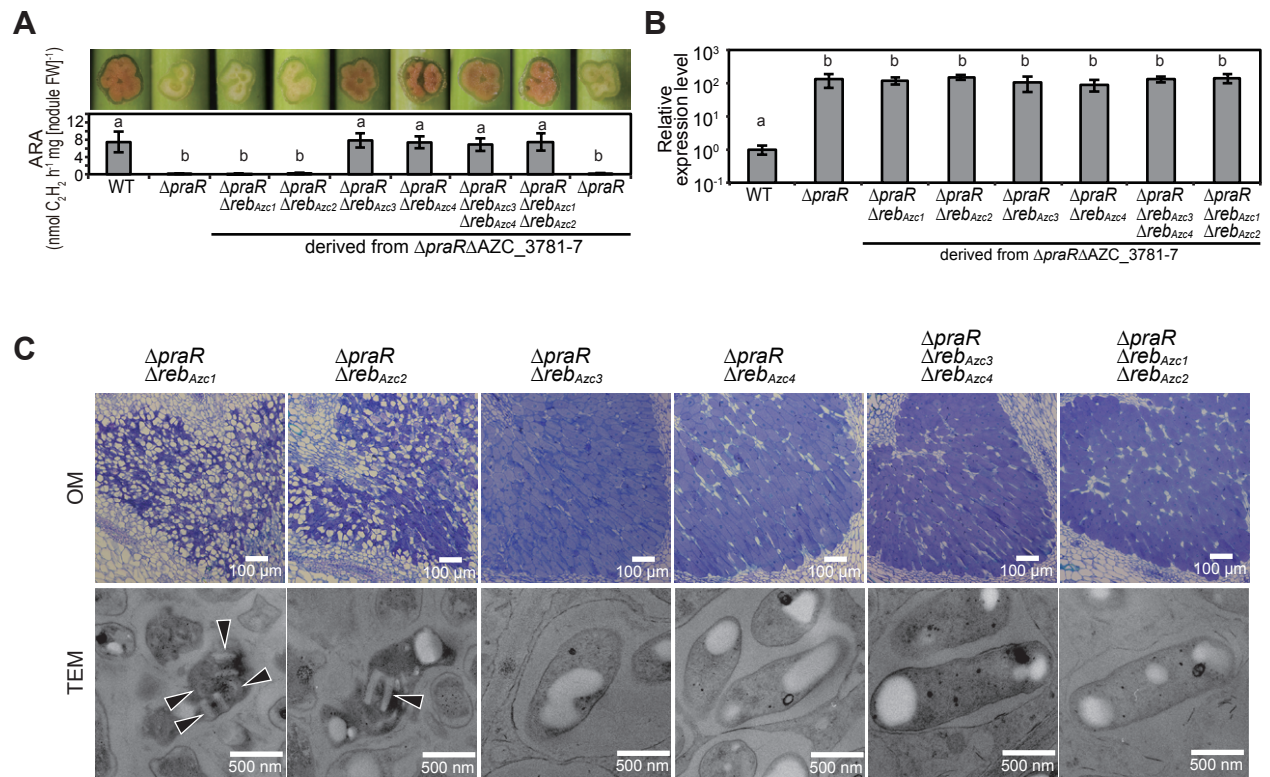

Supplement: FIG S4 [file mbo004173406sf4.pdf]

**A**

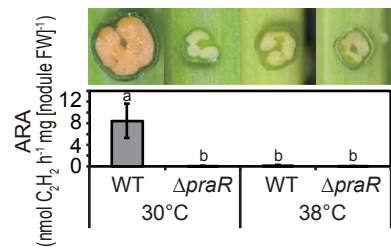

**B**

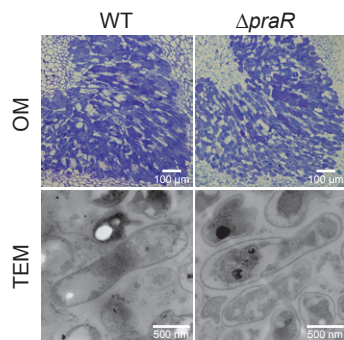

**C**

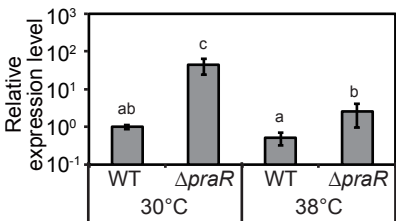

Supplement: FIG S6 [file mbo004173406sf6.pdf]

**A**

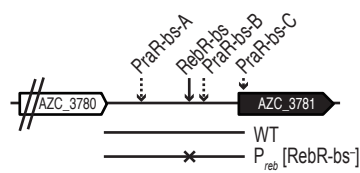

**B**

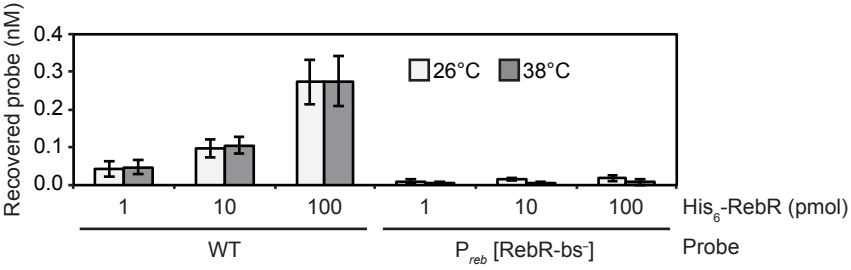

**C**

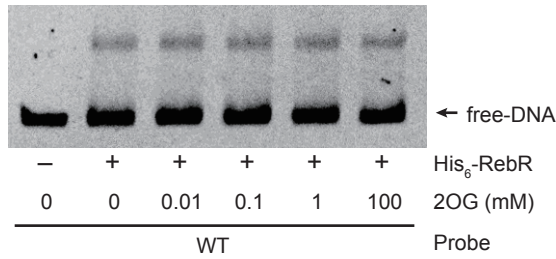

Supplement: FIG S7 [file mbo004173406sf7.pdf]
