# Supplementary material for: Stringent Expression Control of Pathogenic R-body Production in Legume Symbiont Azorhizobium caulinodans
Source: mBio. 2017 Jul 25;8(4):e00715-17. doi: 10.1128/mBio.00715-17 (PMC5527310; doi:10.1128/mBio.00715-17)
Supplement: FIG S5 [file mbo004173406sf5.pdf]

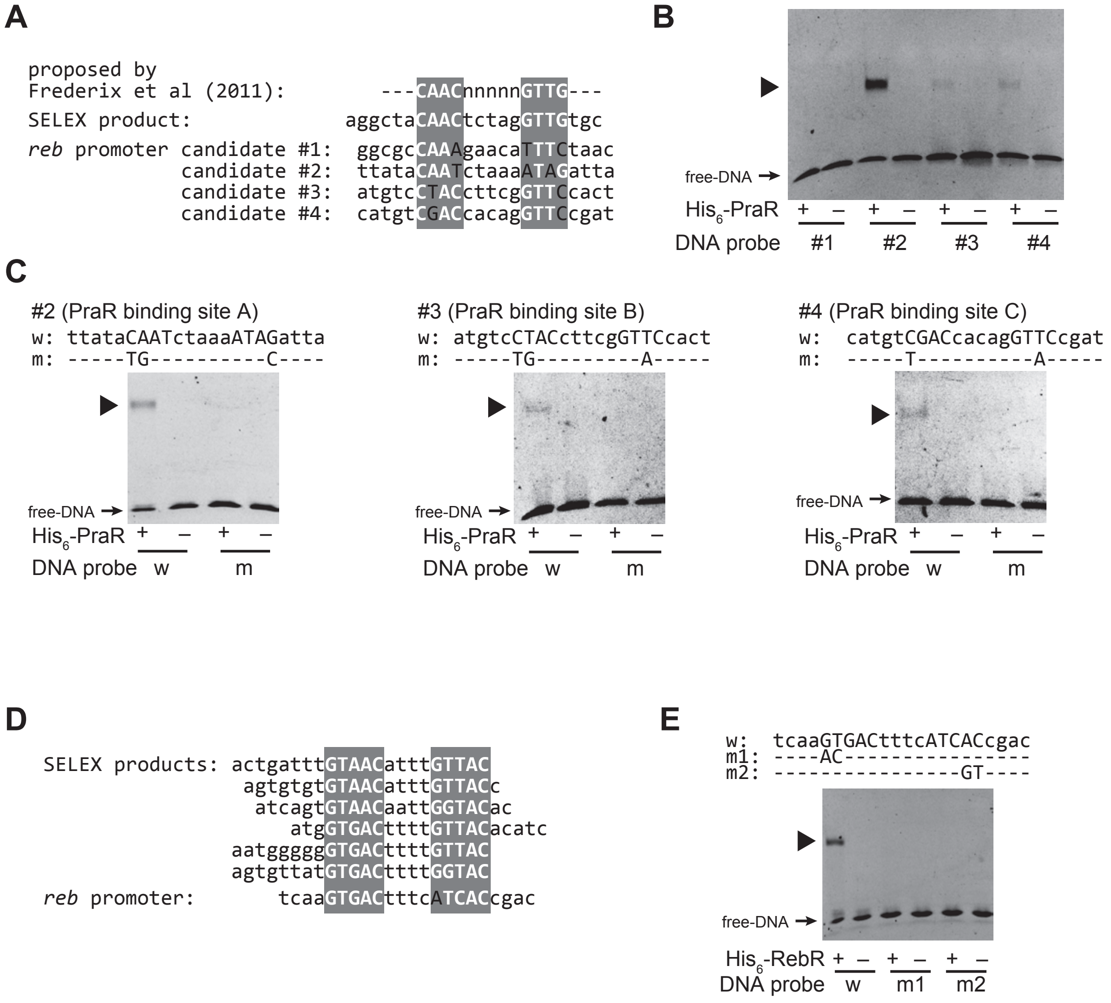
A
proposed by
Frederix et al (2011): ---CAACnnnnnGTTG---
SELEX product: aggctaCAACtctagGTTGtgc
reb promoter candidate #1: ggcgcCAAAgaacaTTTCtaac
candidate #2: ttataCAATctaaaATAGatta
candidate #3: atgtcCTACcttcgGTTCcact
candidate #4: catgtCGACcacagGTTCcgat
B
free-DNA
His6-PraR + – + – + – + –
DNA probe #1 #2 #3 #4
C
#2 (PraR binding site A)
w: ttataCAATctaaaATAGatta
m: -----TG---------C----
free-DNA
His6-PraR + – + –
DNA probe w m
#3 (PraR binding site B)
w: atgtcCTACcttcgGTTCcact
m: -----TG---------A-----
free-DNA
His6-PraR + – + –
DNA probe w m
#4 (PraR binding site C)
w: catgtCGACcacagGTTCcgat
m: -----T----------A-----
free-DNA
His6-PraR + – + –
DNA probe w m
D
SELEX products: actgatttGTAACatttGTTAC
agtgtgtGTAACatttGTTACc
atcagtGTAACaattGGTACac
atgGTGACttttGTTACacatc
aatgggggGTGACttttGTTAC
agtgttatGTGACttttGGTAC
reb promoter: tcaaGTGACtttcATCACcgac
E
w: tcaaGTGACtttcATCACcgac
m1: ----AC----------------
m2: ----------------GT----
free-DNA
His6-RebR + – + – + –
DNA probe w m1 m2
