# Supplementary material for: Stringent Expression Control of Pathogenic R-body Production in Legume Symbiont Azorhizobium caulinodans
Source: mBio. 2017 Jul 25;8(4):e00715-17. doi: 10.1128/mBio.00715-17 (PMC5527310; doi:10.1128/mBio.00715-17)
Supplement: TABLE S1 [file mbo004173406st1.docx]

**Table S1. Bacterial strains and plasmids used in this study**

| **Strain or plasmid** | | **Description** | **Construction method** | **Source or reference** |
| --- | --- | --- | --- | --- |
| ***A. caulinodans*** | | | | |
|  | ORS571 (WT) | Wild-type strain | – | (1) |
|  | Anx7 (∆*praR*) | ORS571 derivative; ∆*praR* | – | (2) |
|  | Anx156 (∆AZC_3781-7) | ORS571 derivative; ∆AZC_3781-7 | – | (2) |
|  | Anx157 (∆*praR*∆AZC_3781-7) | Anx7 derivative; ∆*praR* ∆AZC_3781-7 | – | (2) |
|  | Anx264 (∆AZC_3784) | ORS571 derivative; ∆AZC_3784 | pTAC180 was transconjugated into ORS571 | This study |
|  | Anx265 (∆*praR*∆AZC_3784) | Anx7 derivative; ∆*praR* ∆AZC_3784 | pTAC180 was transconjugated into Anx7 | This study |
|  | Anx266 (∆AZC_3785) | ORS571 derivative; ∆AZC_3785 | pTAC181 was transconjugated into ORS571 | This study |
|  | Anx267 (∆*praR*∆AZC_3785) | Anx7 derivative; ∆*praR* ∆AZC_3785 | pTAC181 was transconjugated into Anx7 | This study |
|  | Anx218 (∆AZC_3787) | ORS571 derivative; ∆AZC_3787 | pTAC131 was transconjugated into ORS571 | This study |
|  | Anx219 (∆*praR*∆AZC_3787) | Anx7 derivative; ∆*praR* ∆AZC_3787 | pTAC131 was transconjugated into Anx7 | This study |
|  | Anx270 (∆*rebR*) | ORS571 derivative; ∆*rebR* | pTAC183 was transconjugated into ORS571 | This study |
|  | Anx271 (∆*praR*∆*rebR*) | Anx7 derivative; ∆*praR* ∆ *rebR* | pTAC183 was transconjugated into Anx7 | This study |
|  | Anx415 (∆*praR*∆*reb_AZC1_*) | Anx157 derivative; complemented with AZC_3781-7 (∆*reb_AZC1_*) | pTAC191 was transconjugated into Anx157 | This study |
|  | Anx416 (∆*praR*∆*reb_AZC2_*) | Anx157 derivative; complemented with AZC_3781-7 (∆*reb_AZC2_*) | pTAC192 was transconjugated into Anx157 | This study |
|  | Anx417 (∆*praR*∆*reb_AZC3_*) | Anx157 derivative; complemented with AZC_3781-7 (∆*reb_AZC3_*) | pTAC193 was transconjugated into Anx157 | This study |
|  | Anx418 (∆*praR*∆*reb_AZC4_*) | Anx157 derivative; complemented with AZC_3781-7 (∆*reb_AZC4_*) | pTAC194 was transconjugated into Anx157 | This study |
|  | Anx419 (∆*praR*∆*reb_AZC3_*∆*reb_AZC4_*) | Anx157 derivative; complemented with AZC_3781-7 (∆*reb_AZC3_*∆*reb_AZC4_*) | pTAC195 was transconjugated into Anx157 | This study |
|  | Anx420 (∆*praR*∆*reb_AZC1_*∆*reb_AZC2_*) | Anx157 derivative; complemented with AZC_3781-7 (∆*reb_AZC1_*∆*reb_AZC2_*) | pTAC196 was transconjugated into Anx157 | This study |
|  | Anx430 (∆*praR*) | Anx157 derivative; complemented with AZC_3781-7 (WT) | pTAC190 was transconjugated into Anx157 | This study |
|  | Anx226 (P*_reb_*[PraR-bs-A^–^]) | ORS571 derivative; base substitutions in the PraR-bs-A on the *reb* promoter | pTAC144 was transconjugated into ORS571 | This study |
|  | Anx233 (P*_reb_*[RebR-bs^–^]) | ORS571 derivative; base substitutions in the RebR-bs on the *reb* promoter | pTAC151 was transconjugated into ORS571 | This study |
|  | Anx234 (P*_reb_*[PraR-bs-A^–^ RebR-bs^–^]) | ORS571 derivative; base substitutions in the PraR-bs-A and the RebR-bs on the *reb* promoter | pTAC152 was transconjugated into ORS571 | This study |
|  | Anx216 (*reb*-*lacZ*) | ORS571 derivative; *reb-lacZ* | pTAC184 was transconjugated into ORS571 | This study |
|  | Anx217 (*reb*-*lacZ*∆*praR*) | Anx7 derivative; *reb-lacZ* ∆*praR* | pTAC184 was transconjugated into Anx7 | This study |
| ***E. coli*** | | | | |
|  | DH5α | FF-,Φ80∆*lacZ*∆M15∆（*lacZYA*-*argF*）U169 *recA endA hsdR supE44 thi gyrA reLAR* | – | (3) |
|  | S17-1 λpir | F-,*thi pro hsdR* [RP4-2 Tc::*Mu* Km::Tn7（Tp Sm）] （λpir） | – | (4) |
|  | BLR-DE3 | F^–^ *ompT* *hsdS*_B_(r_B_^–^ m_B_^–^) *gal* *dcm*(DE3) ∆(*srl-recA*)*306*::Tn*10* (Tet^r^) | – | Novagen |
| **Plasmids** | | | | |
|  | pK18*mobsacB* | Suicide vector | – | (5) |
|  | pUC18 | Cloning vector | – | (6) |
|  | pCold I | Cold shock expression vector | – | Takara-Bio |
|  | pTA-MTL | Suicide vector carrying *lacZ* reporter gene | – | (7) |
|  | pTAC180 | pK18*mobsacB* carrying ∆AZC_3784 fragment | Two fragments amplified from the WT genomic DNA by PCR using primer pairs, Acp634-Acp735 and Acp736-Acp637, were directionally cloned into pK18*mobsacB*. | This study |
|  | pTAC181 | pK18*mobsacB* carrying ∆AZC_3785 fragment | Two fragments amplified from the WT genomic DNA by PCR using primer pairs, Acp634-Acp737 and Acp738-Acp637, were directionally cloned into pK18*mobsacB*. | This study |
|  | pTAC131 | pK18*mobsacB* carrying ∆AZC_3787 fragment | Two fragments amplified from the WT genomic DNA by PCR using primer pairs, Acp638-Acp656 and Acp657-Acp658, were directionally cloned into pK18*mobsacB*. | This study |
|  | pTAC183 | pK18*mobsacB* carrying ∆*rebR* fragment | Two fragments amplified from the WT genomic DNA by PCR using primer pairs, Acp642-Acp643 and Acp732-Acp645, were directionally cloned into pK18*mobsacB*. | This study |
|  | pTAC190 | pK18*mobsacB* carrying WT AZC_3781-7 fragment | A fragment amplified from the WT genomic DNA by PCR using a primer pair, Acp659-Acp715, was cloned into pK18*mobsacB*. | This study |
|  | pTAC191 | pK18*mobsacB* carrying ∆*reb_AZC1_* fragment | *reb_AZC1_* was removed from pTAC190 by inverse PCR using a primer pair, Acp716-Acp717. | This study |
|  | pTAC192 | pK18*mobsacB* carrying ∆*reb_AZC2_* fragment | *reb_AZC2_* was removed from pTAC190 by inverse PCR using a primer pair, Acp718-Acp719. | This study |
|  | pTAC193 | pK18*mobsacB* carrying ∆*reb_AZC3_* fragment | *reb_AZC3_* was removed from pTAC190 by inverse PCR using a primer pair, Acp720-Acp721. | This study |
|  | pTAC194 | pK18*mobsacB* carrying ∆*reb_AZC4_* fragment | *reb_AZC4_* was removed from pTAC190 by inverse PCR using a primer pair, Acp724-Acp725. | This study |
|  | pTAC195 | pK18*mobsacB* carrying ∆*reb_AZC3_* ∆*reb_AZC4_* fragment | *reb_AZC4_* was removed from pTAC193 by inverse PCR using a primer pair, Acp724-Acp725. | This study |
|  | pTAC196 | pK18*mobsacB* carrying ∆*reb_AZC1_* ∆*reb_AZC2_* fragment | *reb_AZC2_* was removed from pTAC191 by inverse PCR using a primer pair, Acp718-Acp719. | This study |
|  | pTAC143 | pK18*mobsacB* carrying WT *reb* promoter | A fragment amplified from the WT genomic DNA by PCR using a primer pair, Acp624-Acp652, was cloned into pK18*mobsacB*. | This study |
|  | pTAC144 | pK18*mobsacB* carrying *reb* promoter fragment with mutation in the PraR binding site A | The PraR-bs-A on pTAC143 was mutated by inverse PCR using a primer pair, Acp693-Acp694. | This study |
|  | pTAC151 | pK18*mobsacB* carrying *reb* promoter fragment with mutation in the RebR binding site | The RebR-bs on pTAC143 was mutated by inverse PCR using a primer pair, Acp699-Acp700. | This study |
|  | pTAC152 | pK18*mobsacB* carrying *reb* promoter fragment with mutations in the PraR binding site A and the RebR binding site | The RebR-bs on pTAC151 was mutated by inverse PCR using a primer pair, Acp699-Acp700. | This study |
|  | pTAC184 | pK18*mobsacB* carrying *reb*-*lacZ* fragment | Two fragments amplified from the WT genomic DNA by PCR using primer pairs, Acp649-Acp740 and Acp651-Acp652, and *lacZ* fragment from pTA-MTL using a primer pair, Tp77-Tp18, were cloned into pK18*mobsacB*. | This study |
|  | pTAC99 | pCold I expressing His_6_-PraR | A fragment amplified from the WT genomic DNA by PCR using a primer pair, Acp375-Acp161, was cloned into pCold I. | This study |
|  | pTAC133 | pCold I expressing His_6_-RebR | A fragment amplified from the WT genomic DNA by PCR using a primer pair, Acp669-Acp670, was cloned into pCold I. | This study |

**References**

1. Dreyfus B, Garcia JL, Gillis M (1988) Characterization of *Azorhizobium caulinodans* gen. nov., sp. nov., a stem-nodulating nitrogen-fixing bacterium isolated from *Sesbania rostrata*. *Int J Syst Bacteriol* 38(1):89–98.

2. Akiba N, Aono T, Toyazaki H, Sato S, Oyaizu H (2010) *phrR*-like gene *praR* of *Azorhizobium caulinodans* ORS571 is essential for symbiosis with *Sesbania rostrata* and is involved in expression of *reb* genes. *Appl Environ Microbiol* 76(11):3475–3485.

3. Grant SG, Jessee J, Bloom FR, Hanahan D (1990) Differential plasmid rescue from transgenic mouse DNAs into *Escherichia coli* methylation-restriction mutants. *Proc Natl Acad Sci USA* 87(12):4645–4649.

4. Simon R, Priefer U, Pühler A (1983) A broad host range mobilization system for *in* *vivo* genetic engineering: Transposon mutagenesis in gram negative bacteria. *Nat Biotechnol* 1(9):784–791.

5. Schäfer A, et al. (1994) Small mobilizable multi-purpose cloning vectors derived from the *Escherichia coli* plasmids pk18 and pk19: selection of defined deletions in the chromosome of *Corynebacterium glutamicum*. *Gene* 145(1):69–73.

6. Yanisch-Perron C, Vieira J, Messing J (1985) Improved M13 phage cloning vectors and host strains: nucleotide sequences of the M13mp18 and pUC19 vectors. *Gene* 33(1):103–119.

7. Iki, Aono, Oyaizu (2007) Evidence for functional differentiation of duplicated *nifH* genes in *Azorhizobium caulinodans*. *FEMS Microbiol Lett* 274(2):173–179.
