# Supplementary material for: Stringent Expression Control of Pathogenic R-body Production in Legume Symbiont Azorhizobium caulinodans
Source: mBio. 2017 Jul 25;8(4):e00715-17. doi: 10.1128/mBio.00715-17 (PMC5527310; doi:10.1128/mBio.00715-17)
Supplement: TABLE S2 [file mbo004173406st2.docx]

**Table S2. Primers used in this study.**

| **Primer** | | **Sequence (5´-3´)** | **Description for underlined sequence** |
| --- | --- | --- | --- |
| **Linearization of pK18*mobsacB*** | | |  |
|  | Tp73 | GGGTACCGAGCTCGAATTCGTAATC |  |
|  | Tp74 | GGGGATCCTCTAGAGTCGACCTGC |  |
| **Linearization of pCold I** | | |  |
|  | Tp78 | ATGCCTACCTTCGATATGATGATG |  |
|  | Tp79 | GTCGACCTGCAGTCTAGATAGGTAATC |  |
| **Linearization of pUC18** | | |  |
|  | Tp61 | AGCTGTTTCCTGTGTGAAATTGTTATCC |  |
|  | Tp62 | GGGAAAACCCTGGCGTTACCCAAC |  |
| **pTAC180** | | |  |
|  | Acp634 | TCGAGCTCGGTACCCGAAGAAACCCCAGAATGCAA | Complement with Tp73 |
|  | Acp735 | CATGACATTCGGCTCCTTC |  |
|  | Acp736 | GAGCCGAATGTCATGTCCAAGATCTGAGGAGGCAAG | Complement with Acp735 |
|  | Acp637 | CTCTAGAGGATCCCCCGGTAGTCCTGGATGCTGAT | Complement with Tp74 |
| **pTAC181** | | |  |
|  | Acp634 | TCGAGCTCGGTACCCGAAGAAACCCCAGAATGCAA | Complement with Tp73 |
|  | Acp737 | CACGCTTGCCTCCTCAGA |  |
|  | Acp738 | GAGGAGGCAAGCGTGCGCTTTCCGAAGTCGAAACT | Complement with Acp737 |
|  | Acp637 | CTCTAGAGGATCCCCCGGTAGTCCTGGATGCTGAT | Complement with Tp74 |
| **pTAC131** | | |  |
|  | Acp638 | TCGAGCTCGGTACCCGCTTTCCGAAGTCGAAACTG | Complement with Tp73 |
|  | Acp656 | CTCGGTGGCGACATTGTTCGGGAAGACGCCATAG | Complement with Acp657 |
|  | Acp657 | AATGTCGCCACCGAGACG |  |
|  | Acp658 | CTCTAGAGGATCCCCGTGGCGACTTCGAAACTCAG | Complement with Tp74 |
| **pTAC183** | | |  |
|  | Acp642 | TCGAGCTCGGTACCCCATCCAGATGCAGATTGTGG | Complement with Tp73 |
|  | Acp643 | AAAGAAGCTGCCAGGATGAA |  |
|  | Acp732 | CCTGGCAGCTTCTTTCAGAACTTCGAACGGTGACG | Complement with Acp643 |
|  | Acp645 | CTCTAGAGGATCCCCTATCTGACGTTCGAGGACGA | Complement with Tp74 |
| **pTAC190** | | |  |
|  | Acp659 | TCGAGCTCGGTACCCCTGCGAAGTTCTGCTGCTC | Complement with Tp73 |
|  | Acp715 | CTCTAGAGGATCCCCGGCATTGTTCACCTTGTCCT | Complement with Tp74 |
| **pTAC191** | | |  |
|  | Acp716 | GCGTTCTCAAGGCACTCACCACGAAA | Complement with Acp717 |
|  | Acp717 | GTGCCTTGAGAACGCTCTCGCTGCT | Complement with Acp716 |
| **pTAC192, pTAC196** | | |  |
|  | Acp718 | GAACGAGGATCGCCATCGTCCAGATG | Complement with Acp719 |
|  | Acp719 | TGGCGATCCTCGTTCCGGTTTGCAT | Complement with Acp718 |
| **pTAC193** | | | |
|  | Acp720 | CAGTGTCCTGTCCACGCCCTGAAAT | Complement with Acp721 |
|  | Acp721 | GTGGACAGGACACTGGTGCTGTTGTTGG | Complement with Acp720 |
| **pTAC194, pTAC195** | | |  |
|  | Acp724 | AAACGAGGAACACCTGACACGCAACG | Complement with Acp725 |
|  | Acp725 | AGGTGTTCCTCGTTTCTTCGGTTAGGTC | Complement with Acp724 |
| **pTAC143** | | | |
|  | Acp624 | TCGAGCTCGGTACCCCGGCCTTCGGTTATGACGAC | Complement with Tp73 |
|  | Acp652 | CTCTAGAGGATCCCCAAATTCTGGCGGCCCGTTAT | Complement with Tp74 |
| **pTAC144** | | | |
|  | Acp693 | ATATGGTCTAGAATAGATTAAATAGCTAAC | Complement with Acp694 |
|  | Acp694 | TATTCTAGACCATATAAATTGTTAGAAATG | Complement with Acp693 |
| **pTAC151, pTAC152** | | |  |
|  | Acp699 | TCAAACGAATTCCATCACCGACGCATTTGG | Complement with Acp700 |
|  | Acp700 | ATGGAATTCGTTTGAAGGAAAATATTCGCAGA | Complement with Acp699 |
| **pTAC184** | | |  |
|  | Acp649 | TCGAGCTCGGTACCCGCACCCCTCCCCCTCAAG | Complement with Tp73 |
|  | Acp740 | AATGCCGCCTTAGCACGTCACCGTTCGAAGTTCTG | Complement with Tp77 |
|  | Acp651 | TTTCGCGTAAGGAAACGGTGACGGGGAAGAGGAA | Complement with Tp18 |
|  | Acp652 | CTCTAGAGGATCCCCAAATTCTGGCGGCCCGTTAT | Complement with Tp74 |
|  | Tp77 | TGCTAAGGCGGCATTTTAAC |  |
|  | Tp18 | TTTCCTTACGCGAAATACGG |  |
| **pTAC99** | | |  |
|  | Acp375 | CGGGATCCGTCAAGAAGGCGCCGAAC | *Bam*H I site |
|  | Acp161 | GCTCTAGAAAGCGTTCGTCAAATCGAA | *Xba* I site |
| **pTAC133** | | |  |
|  | Acp669 | ATCGAAGGTAGGCATGCACCCCTCCCCCTCAAG | Complement with Acp670 |
|  | Acp670 | AGACTGCAGGTCGACCGTCACCGTTCGAAGTTCTGC | Complement with Acp669 |
| **RT-PCR** | | |  |
|  | P1 | GATCTGCACCCGCTCTCTAC |  |
|  | P2 | ATTTCGTGGTGAGTGCCTTC |  |
|  | P3 | AACCATTGCAACTCCAGAGG |  |
|  | P4 | ATTTCAGGGCGTGGACAG |  |
|  | P5 | CCAACAACAGCACCAGTGTC |  |
|  | P6 | GCTGTCCTCGTAGGTGCAAT |  |
|  | P7 | GCAAGGACCTAACCGAAGAA |  |
|  | P8 | AAGCCCGGATAGAGGTTGAT |  |
|  | P9 | TTCATGTTCTCGTCCCTCCT |  |
|  | P10 | CCTCGGTGACGAAATAGAGC |  |
|  | P11 | CCTGTTCCAGGGGCTCAC |  |
|  | P12 | GCCCCGAGGAATATTACGAT |  |
|  | P13 | TGTCCTACCTTCGGTTCCAC |  |
| **Primer extension** | | |  |
|  | P14_FITC* | TCGGAACCTGTGGTCGACAT |  |
| **Quantitative RT-PCR for the *reb* operon** | | |  |
|  | Acp326 | CATTGAGGTTGTGCTGGTTC |  |
|  | Acp714 | GAGAACGCTCTCGCTGCT |  |
| **Quantitative RT-PCR for 16SrRNA** | | |  |
|  | Tp35 | ACGGATTTCTTCCAGCAATG |  |
|  | Tp36 | ACCGGCAGTCCCTTTAGAGT |  |
| **SELEX** | | |  |
|  | M13F | CGCCAGGGTTTTCCCAGTCACGAC |  |
|  | M13R | ACACAGGAAACAGCTATGACCATG |  |
| **EMSA** | | |  |
|  | Acp646 | TAAAACAGGGCGTGAAACG |  |
|  | Acp702 | AGGAAACAGCTATGACCATGCCACGCTATCGGTGATGG | M13 Reverse sequence |
|  | M13R_FITC* | ACACAGGAAACAGCTATGACCATG |  |
| **Quantitative PCR for dsDNA probe corresponding to *reb* promoter** | | |  |
|  | Acp629 | TGTCCTACCTTCGGTTCCAC |  |
|  | Acp892 | TCGGAACCTGTGGTCGACAT |  |

* FITC-labeled at 5´-end
